# A method to produce a flexible and customized fuel models dataset

**DOI:** 10.1016/j.mex.2023.102218

**Published:** 2023-05-19

**Authors:** A.C.L. Sá, A. Benali, B.A. Aparicio, C. Bruni, C. Mota, J.M.C. Pereira, P.M. Fernandes

**Affiliations:** a Centro de Estudos Florestais, Instituto Superior de Agronomia, Universidade de Lisboa, Tapada da Ajuda, Lisboa 1349-017, Portugal; b Autoridade Nacional de Emergência e Proteção Civil, Avenida do Forte, Carnaxide 2794-112, Portugal; c ForestWISE - Laboratório Colaborativo para a Gestão Integrada da Floresta e do Fogo, Quinta de Prados, Vila Real 5001-801, Portugal; d Centro de Investigação e de Tecnologias Agroambientais e Biológicas, Universidade de Trás-os-Montes e Alto Douro, Quinta de Prados, Vila Real 5000-801, Portugal

**Keywords:** Land cover, Fire-atlas, Burned areas, Time since last fire, Spectral vegetation indexes, Satellite data, Fuel models, Expert-based knowledge, Flexible approach, Automatic updates, FUMOD: updated fuel models gridded dataset

## Abstract

Simulation of vegetation fires very often resorts to fire-behavior models that need fuel models as input. The lack of fuel models is a common problem for researchers and fire managers because its quality depends on the quality/availability of data. In this study we present a method that combines expert- and research-based knowledge with several sources of data (e.g. satellite and fieldwork) to produce customized fuel models maps. Fuel model classes are assigned to land cover types to produce a basemap, which is then updated using empirical and user-defined rules. This method produces a map of surface fuel models as detailed as possible. It is reproducible, and its flexibility relies on juxtaposing independent spatial datasets, depending on their quality or availability. This method is developed in a ModelBuilder/ArcGis toolbox named FUMOD that integrates ten sub-models. FUMOD has been used to map the Portuguese annual fuel models grids since 2019, supporting regional fire risk assessments and suppression decisions. Datasets, models and supplementary files are available in a repository (https://github.com/anasa30/PT_FuelModels).

• FUMOD is a flexible toolbox with ten sub-models included that maps updated Portuguese fuel models.

## Graphical abstract

**Figure:**
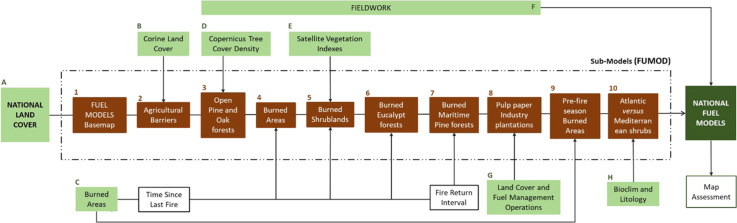

Specifications table

**Table:** 

Subject area:	Agricultural and biological sciences
More specific subject area:	Vegetation data and fire spread modelling
Name of your method:	FUMOD: updated fuel models gridded dataset
Name and reference of original method:	N.A.
Resource availability:	Portuguese Land Cover (COS2018): https://snig.dgterritorio.gov.pt/rndg/srv/por/catalog.search#/search?anysnig=COS&fast=index (last access: 26^th^ December 2022) Portuguese Fire-atlas (1975-2021): https://geocatalogo.icnf.pt/catalogo_tema5.html (last access: 26^th^ December 2022) Corine Land Cover (CLC2018): https://land.copernicus.eu/pan-european/corine-land-cover/clc2018 (last access: 26^th^ December 2022) S2 (Sentinel 2, Level 2A): https://scihub.copernicus.eu/dhus/#/home (last access: 26^th^ December 2022) Tree cover density (TCD): https://land.copernicus.eu/pan-european/high-resolution-layers/forests/tree-cover-density (last access: 26^th^ December 2022) Bioclimatic and Lithologic data (BIOLIT): https://home.isa.utl.pt/~tmh/aboutme/Informacao_bioclimatologica.html (last access: 26^th^ December 2022) ArcGIS survey 123 application: https://play.google.com/store/apps/details?id=com.esri.survey123 (last access: 26^th^ December 2022) Fuel Models survey: https://ulisboa.maps.arcgis.com/apps/dashboards/64918865d7ba4503bce60392531947a0 (last access: 26^th^ December 2022)

## Method details

There has been recent efforts within the scientific community to produce datasets of fuel models given their key role in fire simulations and thus on wildfire management (e.g., [1], [2], [3]). It is well supported by researchers that the main mechanisms driving fire spread and behavior are fire ignitions, meteorology, topography and fuels, with relative contributions depending on ecosystem type and with scale-dependent effects (e.g., [4], [5], [6], [7]). The relationship between fuels composition and structure and fire behavior estimates enhances the importance of accurately mapping fuel models. This is evident in studies of the impact that fuel treatments have in decreasing wildfire intensity [8] or when investigating the reduction of errors in simulated rate of spread [9]. In several fire spread modelling systems (e.g. [10,11]) input data of fuels is supplied by a grid of fuel models [12,13].

The use of custom fuel models developed for heterogeneous landscapes of Mediterranean regions has been unequivocally proved to increase simulation accuracy [9,^14^,15]. Some efforts to produce customized fuel models datasets have relied for example on fuel inventory data [16] or experimental fire data as it is shown in one of the first attempts to develop fuel models in Turkey [3]. Mapping fuel models is a challenging task because of vegetation complexity and dynamics. Vegetation depends on the interaction of climate, soils, topography and anthropic factors, whose gradients and variability in Portugal affect the spatial variability of fuels. Fuel models aggregate a set of characteristics that represent the fuel complex for fire behavior predictions. Fuel models summarize what is commonly defined as “fuelbed”, the entire live and dead surface fuel complex, which includes litter, slash, grasses and shrubs [17]. Canopy (overstorey) fuel characteristics required to simulate crown fire behavior (canopy base height, stand height, canopy bulk density) are not addressed in this work.

The sources of error and uncertainty in mapping fuel models are large, highlighting the importance of multiple sources of data and approaches to represent them more accurately (for a review see [18]). Nonetheless, the importance of having a flexible approach that combines different sources of data for fuel models classification was previously found by Fernandes [2]. For example, vegetation regrowth after a disturbance (e.g. fire, disease, tree harvesting) can be described using different methods and datasets depending or their availability and quality. This flexibility is especially relevant when fire spread simulations are run in near-real-time to support regional fire suppression decisions. However, when considering the production of a pan-European fuel models map, there must be a standardization of the methodology, the data used, and the maps produced. Also, when using different years of fuel data to calibrate a fire spread simulation system, it is required that fuel models maps have been produced using the same methodological approach.

In this work we propose a fuel model mapping method that relies on combining several sources of data to improve the quality of a final fuel model classes map. One of the main advantages is the method flexibility to incorporate other sources of data, depending on their availability, the scale analysis and objectives. This is a framework developed for the Portuguese case study, which relies on the national existing land cover mapping and a fire-atlas of burned area perimeters from 1975 to 2021. The method consists in the integration of ten sub-models in a full model entitled FUMOD (updated fuel models dataset).

## Datasets

Datasets used in the method development are described in Table 1. All data were compiled to a regular 100 m cell size grid, and in the UTM-29N projection system. Datasets F and G correspond to field data and private data from the two main Portuguese pulp industry companies, respectively. In dataset F, data is organized in a geodatabase with polygon features and photos of the surface fuel models collected in the field using the ArcGIS Survey 123 application. These data support the assessment of fuel models maps and the improvement/definition of rules used in the ten sub-models of FUMOD. Dataset G comprises of land cover data from eucalypt pulp paper industry, namely eucalypt plantations, other land cover types, and information regarding fuel reduction treatments and forest harvesting plans. Properties’ information is harmonized and compiled in a shapefile, which is then converted to a 100 m cell grid of fuel models.

**Table 1:** Datasets that integrate the FUMOD model to obtain the national updated fuel model classification.

Dataset	Description	Reference
(A) Land Cover (COS2018)	Portuguese Land Cover map for 2018, with a minimum mapping unit of 1 ha. A raster grid was derived with the spatial resolution of 100 meters.	[49]
(B) Corine Land Cover (CLC2018)	European Land Cover map for 2018 with a minimum mapping unit of 25 hectares.	[50]
(C) National Fire-Atlas	Portuguese burned area perimeters with a minimum mapping unit of 5 hectares, from 1975 to 2021.	[51]
(D) Tree Cover Density (TCD2018)	Copernicus Tree Cover Density (TCD) 2018 High Resolution Layer with spatial resolution of 100 meters.	[45]
(E) Satellite S-2 Vegetation Indexes	Sentinel-2 Level-2a surface reflectance Data. One-month mean reflectance composites were used to calculate NDVI and SAVI spectral vegetation indexes at the spatial resolution of 100 meters. Spectral indexes were calculated for the main fire season from June to September, and for the years between 2018 and 2021.	[52]
(F) Fieldwork	Data on fuel models classes collected using the ArcGIS Survey 123 app via mobile device. Fieldwork done in 2021 and 2022.	-
(G) Pulp industry land cover	Land cover data for eucalypt pulp industry plantations, and silvicultural management operations (private data). Annual data for the years between 2019 and 2022. Data was shared in vector shapefile format.	-
(H) Bioclimatic and lithological map (BIOLIT)	Map of the spatial distribution of Atlantic *versus* Mediterranean forest and shrublands communities, which resulted from the combination of national annual ombrothermic index and soil parent material types.	[53,54]

The Portuguese fuel models [19] are organized in three groups: F (litter); M (mixed: litter and understory vegetation); and V (understory vegetation, shrubland or grassland). There are 18 fuel model classes whose description is presented in Table S1. To account for other surface fuel model classes not represented in the national classification, we added NFFL fuel models [20], namely to classify burned areas with high pine regeneration density; and another typology of fuel models specifically developed for the Center region of Portugal [21], to represent downed and dead woody slash from coppicing in eucalypt forest plantations. Both cases are also presented in Table S1. Portuguese fuel models’ parameters are described in Table S2.

## Fieldwork data

Fieldwork is an important source of reference data. The heterogeneity of the spatial distribution of land use/land cover types and surface fuels makes the assignment of fuel models a challenging task, with different sources of error and uncertainty. For example, the perceptions of fire behavior in the fuelbed and spatial variability of fuel types can be different among users, which can lead to different fuel model classes assignment; or the possibility of assigning the same fuelbed to different vegetation types increases uncertainty regarding the fuel model that best describes fire behavior. To support the definition of rules developed in the FUMOD, a classification survey was developed using the ArcGIS Survey 123 application. The application is free but requires registration. The manual (currently there is only a Portuguese version) is available as a supplementary file in the repository.

**Fig. 1:**
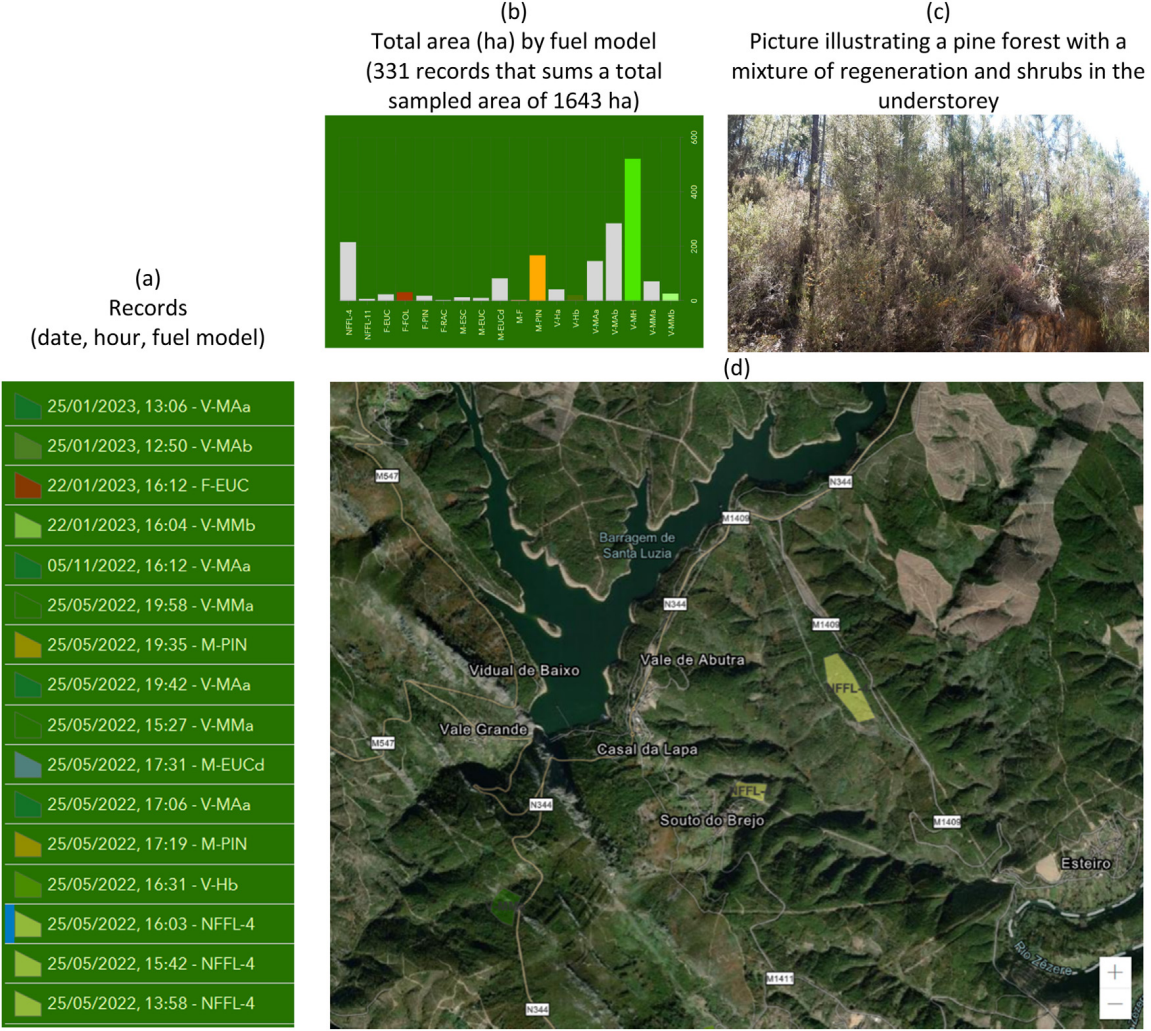
Example extracted from the ArcGIS Survey 123 fuel models application, which includes the list of the total number of samples collected (331) with the identification of data and time, and the corresponding surface fuel model code (a); the total area (ha) of each fuel model class collected (b); a picture taken to illustrate a pine forest area with dense natural regeneration (c) where the NFFL-4 fuel model was assigned as shown in the yellow polygon overlaid in the Google Earth image (d).

The survey protocol includes a sequence of steps to be followed in the field. First, the user needs to find in a previously selected land cover type an area where surface fuel composition is homogeneous and determine what is the main fuel layer(s) responsible for the spread of a fire. In the application, the user is then asked to digitize on screen over a google earth map, a polygon representing the area. Next, a set of IF-THEN-ELSE questions (supported by classification trees, see Fig. S2) where the user selects the answer according to what is observed in the surface fuels, ends with a suggestion of a fuel model and examples of photos to help the decision to accept or reject the proposed fuel model class. This step is important to detect incorrect answers. If accepted, the user is asked to take four photos in the direction of each of the cardinal points. If rejected, the survey restarts until a new decision by the user. The questions in the survey are simple and based on the fuelbed composition, cover and structure (such as shrub fuel depth or litter compactness), according to the vegetation type of the area (Fig. S2). The inventory depends on the experience and visual recognition of the area, without the need of any quantitative measurement. All these steps can be done in offline internet mode. All data is organized in a dashboard with several graphs that summarize and illustrate the data collected. Data can be exported to a geodatabase that can be displayed and manipulated in any GIS program. Fig. 1 shows some of the information in the app (Fig. 1a,b) and an example of an area of natural regeneration of pine (Fig. 1c) and the corresponding polygon with the fuel model assigned (Fig. 1d).

**Fig. 2:**
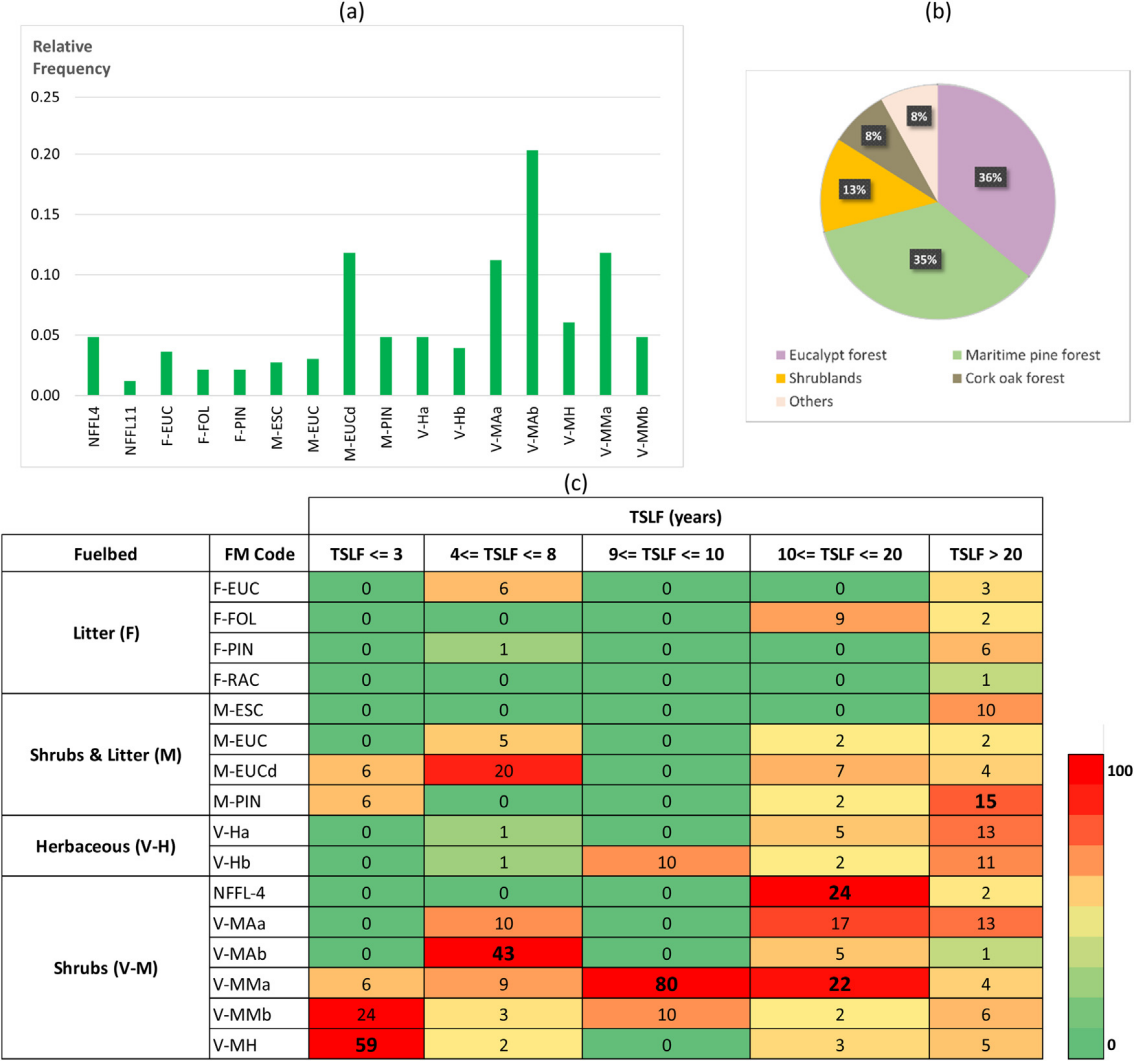
Frequency distributions of fuel model classes (a); percentage of vegetation cover types (b); and color heat (legend with colors distributed in deciles) table of the distribution in % of the fuel models by time since last fire (TSLF), considering the 331 records (for a description of fuel model codes (FM Code) see Fig.S1). For example, 59 % of the areas that burned less than 3 years ago had the V-MH fuel model class assigned.

Fieldwork was carried out in the years of 2021 and 2022 by researchers, foresters, and fire personnel. Time and place of inventories were dependent on people availability, and frequently during no longer than 2-3 days of field work. The most representative fire-prone land cover types where misclassification of fuel models can produce larger impacts in simulated fire spread and behavior estimates were selected for collecting fuel models. At the moment, the survey database comprises 331 records but the goal is to continue collecting data to increase and improve data usage. The most frequent fuel model classes (Fig. 2a) respect to the shrub fuel layer, either in shrublands or in the understory. Approximately 80 % of the land cover types (Fig. 2b) are shrublands, pine forests and eucalypt forests.

The observed dataset of fuel models has been contributing to increased knowledge on the correspondence between the most representative fuel model classes and land cover types, and vegetation recovery in burned areas. Information regarding fuel age (time since fire), required to build some of the FUMOD rules, was also considered to target fieldwork areas (Fig. 2c). Time since last fire (TSLF) was used to assign different surface fuel models: for example, in tall *versus* short shrubs, in recently burned areas, and in high-density naturally-regenerated pine stands. We should be aware that there are some sources of errors affecting fuel model classification, as for example, the user perception of what is the expected fire behavior in the area, dataset errors, surface fuels heterogeneity within and between vegetation patches, the influence of non-fuel factors in fire behavior, and user expertise to assign a fuel model class to a land cover type.

## FUMOD

This work proposes a method that consists in producing a customized and updated fuel models map (FMM) from a fuel model basemap (FMB) obtained by assigning fuel model classes to main land cover types. Different intermediate FM maps are added to update the FMB, mainly accounting for vegetation recovery in burned areas, agricultural barriers, changes in surface fuels in open forests, and changes in fuels due to information of forest management operations in eucalypt industry plantations. This approach is flexible and can be automatically reproduced, enabling the inclusion or exclusion of layers from different variables, depending on their availability and/or data quality. The method has been applied to produce the Portuguese mainland annual FM maps from 2019 to 2022 and used as input data to simulate fire spread for operational fire suppression activities and in several research studies [22], [23], [24], [25], [26], [27], [28], [29].

**Fig. 3:**
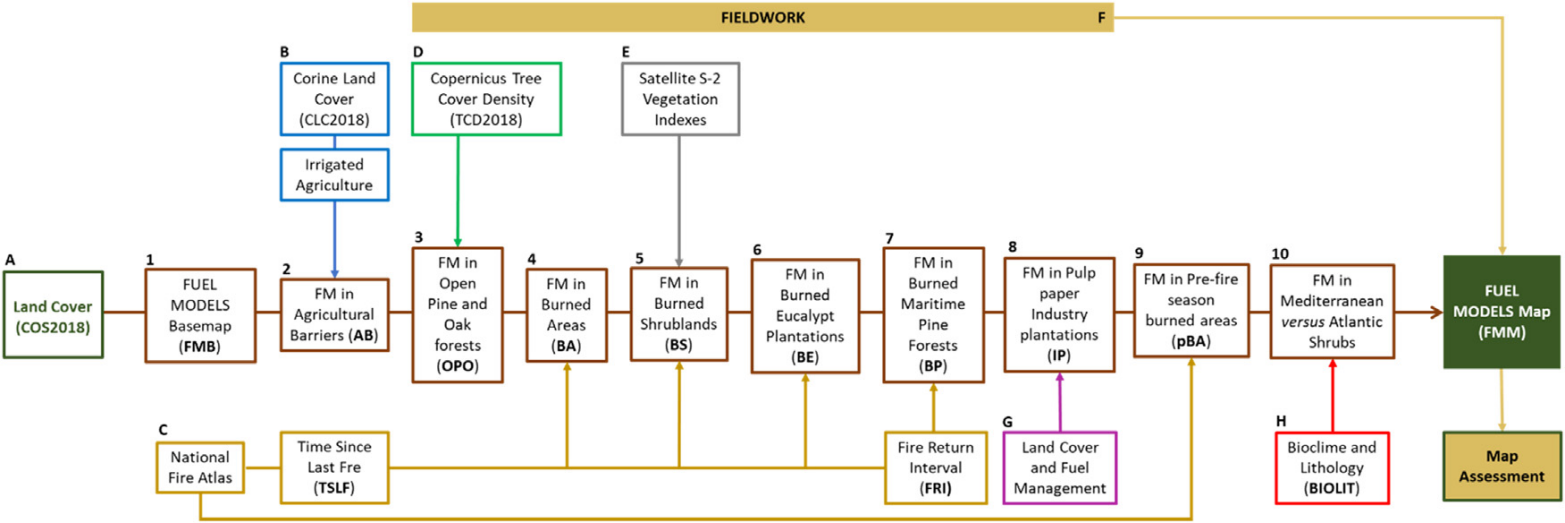
Flowchart representing the proposed FUMOD method, composed by a sequence of the 10 sub-models (SM) and eight datasets (labeled from A to H) to automatically produce the final updated fuel models map (FMM). See Table 1 for the description of each dataset, and the next section for the description of each SM.

The Portuguese FM map is produced annually at the beginning (typically in June) of the main fire season (from June to October). It covers an area of ca. 90,000 km^2^, with a grid cell size of 100 m. Each FM map results from the combination of expert knowledge, literature, satellite-derived datasets, empirical and user-defined rules and of fieldwork data. Fig. 3 shows the full model (FUMOD) that integrates 10 sub-models (SM), and the datasets labeled from A to H that are necessary to produce the final updated FMM dataset. The method is implemented in ModelBuilder/ArcGis program by creating a toolbox that includes the developed sub-models and geodatabases, all shared for the scientific and operational communities in the repository (https://github.com/anasa30/PT_FuelModels). Fig. 3 shows in detail the flowchart of the FUMOD.

## Sub-models (SM)

### SM1: Fuel models basemap (FMB) - uses dataset A (COS2018)

The FMB results from assigning the Portuguese fuel model classes to land cover types of the Portuguese Land Cover dataset (COS2018). Table 2 describes the assignments considered to generate the FMB from authors expertise on the relationship between vegetation types and fire behavior characteristics. The authors have experience and proven scientific knowledge on fuel model development, including validation with real case fires [2,^15^,19], and in fire simulation and the relationships between fire descriptors, vegetation types and fuels (e.g., [25,^28^,30]). Fire spread barriers (e.g., water, infrastructures, built-up areas) and likely unburnable fuels (e.g., irrigated agriculture) are coded with 98 (hereafter denoted “unburnable”). The fuel model class assignment does not consider the litter group fuel models, since it is not possible to differentiate whether the understory is dominated by litter or by a mixture of litter and vegetation based only on land cover information. Therefore, we assume this potential misclassification whereas it is preferable to overestimate potential fire behavior than underestimate it.

**Table 2:** Fuel model class assignment to the Portuguese Land Cover types (COS2018).

### SM2: Agricultural barriers - uses datasets B (CLC2018)

The CORINE Land Cover dataset for Portugal for the reference year 2018 (CLC2018) is used to create the gridded mask of irrigated agriculture, which is then assigned to unburnable fuels. Despite the lower spatial resolution of this dataset compared with COS2018, the last has a single class that mixes irrigated and rainfed land cover types.

### SM3: Fuel models in unburned maritime pine and oak open forests - uses dataset C (fire-atlas), E (tree cover density) and F (fieldwork)

The product of Tree Cover Density (TCD) is used to select open forests (defined as having TCD below 40%) whereas surface fuels may be dominated by shrubs. Thus, the next rules define the FM assigned to: a) maritime (*Pinus pinaster*) and stone pine (*Pinus pinea*) forests; and b) cork (*Quercus suber*) and holm (*Quercus rotundifolia*) woodlands.

(a) IF TCD <40% THEN FM=234/237 ELSE FM=227 (for pine forests; depending whether shrubs are Atlantic (234) or Mediterranean (237)).
(b) IF TCD <10% THEN FM=232 ELSE (IF (TCD ≥10% AND TCD<40%) THEN FM=234/237 ELSE FM=222)) (for woodlands; depending whether shrubs are Atlantic (234) or Mediterranean (237)).

### SM4: Fuel models in burned areas - uses dataset C (fire-atlas), D (spectral indexes) and F (fieldwork)

For all the burned area perimeters (larger than 5 ha) from 2018 to 2021 (in DATASET C), there is information on the time since last fire (TSLF), the year of the last fire (AUF), the number of times burned (NX) and the mean fire return interval (FRI). Mean FRI is calculated as the ratio between the number of years included in the fire-atlas and the number of times each cell burns (here 48 years for the FMM of 2022). TSLF and FRI are rasterized to 100 m cell size grids.

**Fig. 4:**
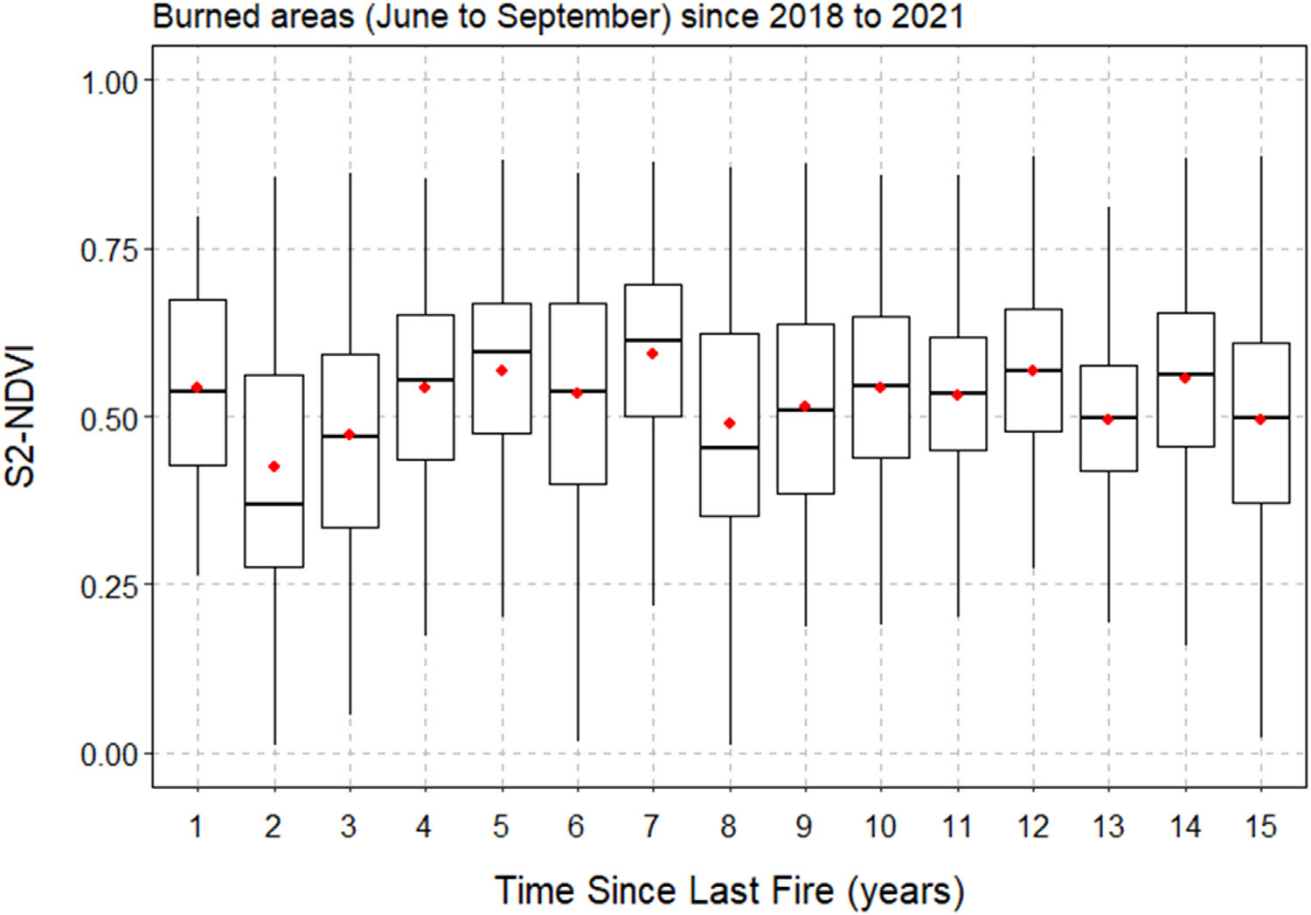
Comparative box-plots of NDVI vegetation index as a function of burned area age (TSLF). NDVI values were extracted from mean monthly composites of Sentinel-2 reflectance data for the month before the date of fire occurrence. Outliers were removed from the plot. Red dots and horizontal lines inside the box represent the mean and median, respectively. The upper and lower box limits represent the 25^th^ and 75^th^ percentiles and the whiskers extend to 10^th^ and 90^th^ percentiles.

The Normalized Difference Vegetation Index (NDVI) is widely used to quantify vegetation greenness and to capture vegetation density changes over time [31], namely those associated with vegetation regrowth in recently burned areas. Such potential may help the assignment of low fuel loading models in these areas. In this SM, the NDVI is calculated using the red and near infra-red bands from mean surface reflectance composites of Sentinel-2 imagery. Composites are calculated for the previous month of the fire date, for all fires that occurred during the main fire season (from June to September). Next, the NDVI and the TSLF are extracted to a database. The analysis of the relationship between burned area age and the mean NDVI is shown in Fig. 4.

Fig. 4 shows an overall increase in vegetation greenness up to 5 years, due to fuel build-up with time. Among other factors, climate, fire frequency and elevation affect vegetation recovery [32]. However, these variables were not included in the analysis to avoid further complexity in the definition of SM rules. We combined information from Fig. 4 with fieldwork data (Fig. 2c) to build the rules shown in Table 3. Therefore, as indicated in Fig. 2c, for visited burned areas up to 3-year-old, 59 % were classified as sparse vegetation (fuel model V-MH), characterized by sparse low shrubs interspersed sometimes with grasses. Older burned areas have an increase of low shrubs cover as fuels build-up with age.

**Table 3:** Fuel Model (FM) class assignment in burned areas with less than five years. TSLF = Time since last fire.

### SM5: Fuel models in burned shrublands - uses dataset C (fire-atlas) and D (spectral indexes)

For shrublands burned from 2018 to 2021, the TSLF grid is used to select those up to 10-years-old to account for the temporal and spatial variability in shrub biomass cover and accumulation, and thus the heterogeneity of surface fuel models. There is a large uncertainty regarding when there is a transition from low to tall shrubs, with the corresponding changes in fuel accumulation. Based on previous work [33,34] there is a large variability in the relationship between shrub biomass and shrub age. For shrub ages of 9 and 10 years, the model estimates similar average biomass [33]. Fig. 4 shows a decrease in the NDVI in 8-year-old shrublands afterwards. We performed a Wilcox rank sum test to compare the NDVI medians from shrublands of 7 years old and from shrublands older than 7 years, and the difference between medians are statistically different from 0, at the level of confidence of 95 %. This result may suggest a tipping point at the age of eight. This can be related with structurally mature vegetation, with less productive/healthy phenology and more dead fuel, thus lower greenness. However, besides observation of these areas in the fieldwork, we could not find any support in the literature. Table 3 shows the FM classes assigned to shrubs with ages between 6 and 10.

**Fig. 5:**
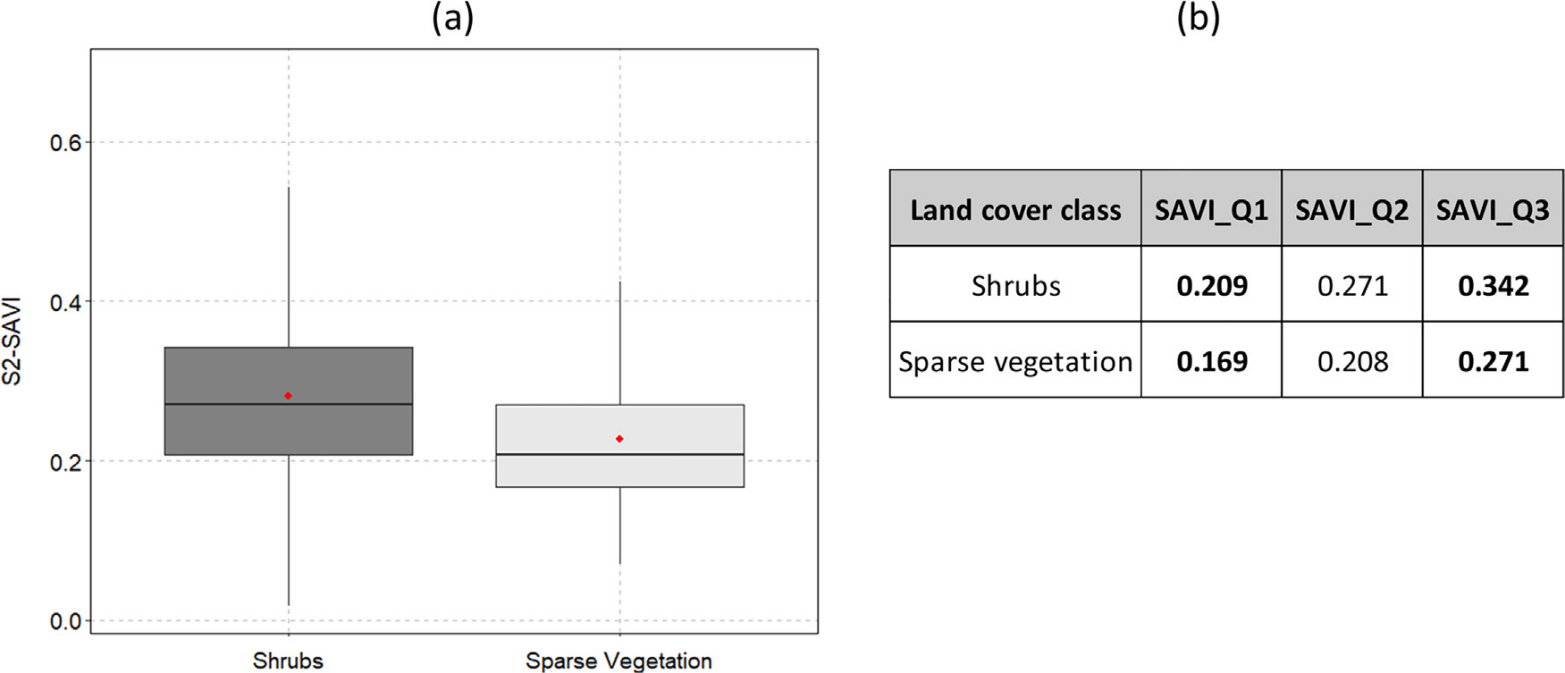
Comparison between the distribution of Sentinel-2 derived SAVI vegetation index in shrubs and sparse vegetation land cover types. SAVI values were extracted from monthly composites of Sentinel-2 mean surface reflectance for the month before fire occurrence. Red dots and horizontal lines inside the box represent the mean and median, respectively. The upper and lower box limits represent the 25^th^ and 75^th^ percentiles and the whiskers extend to 10^th^ and 90^th^ percentiles.

The spatial heterogeneity of vegetation can be high in burned areas. Satellite data and spectral vegetation indexes can be used to assess the amount and healthy status of vegetation [35]. The Sentinel-2 monthly composites of mean reflectance used in SM4 were also used to calculate the Soil-Adjusted Vegetation Index (SAVI) [36]. The SAVI vegetation index corrects the NDVI for the influence of soil brightness in sparsely vegetated areas. The distribution of the SAVI in shrublands and sparsely vegetated areas and the corresponding quantile values are shown in Fig. 5. For example, consider a burned area from the previous year, thus unlikely to burn (assigned FM 98, Table 3). If there are cells with SAVI values above 0.342 (75^th^ percentile) within the burned area perimeter, then those areas may consist of low shrubs (rule a). Conversely, very low SAVI values (below the 25^th^ percentile) in areas of tall or low shrubs, likely those areas are sparsely vegetated (rule b). Middle values of SAVI were only selected in areas classified as unburnable to change them to a low fuel load fuel model class (rule c). Therefore the rules used to update fuel models inside burned shrublands are:

IF SAVI≥0.342 & FM=98 THEN FM=234/237 (depending whether shrubs are Atlantic (234) or Mediterranean (237))

(a) IF SAVI ≤0.209 & (FM=233/236 OR FM=234/237) THEN FM=235 (depending whether shrubs are Atlantic (233) or Mediterranean (236))
(b) IF 0.169<SAVI<0.271 & FM=98 THEN FM=235

**Fig. 6:**
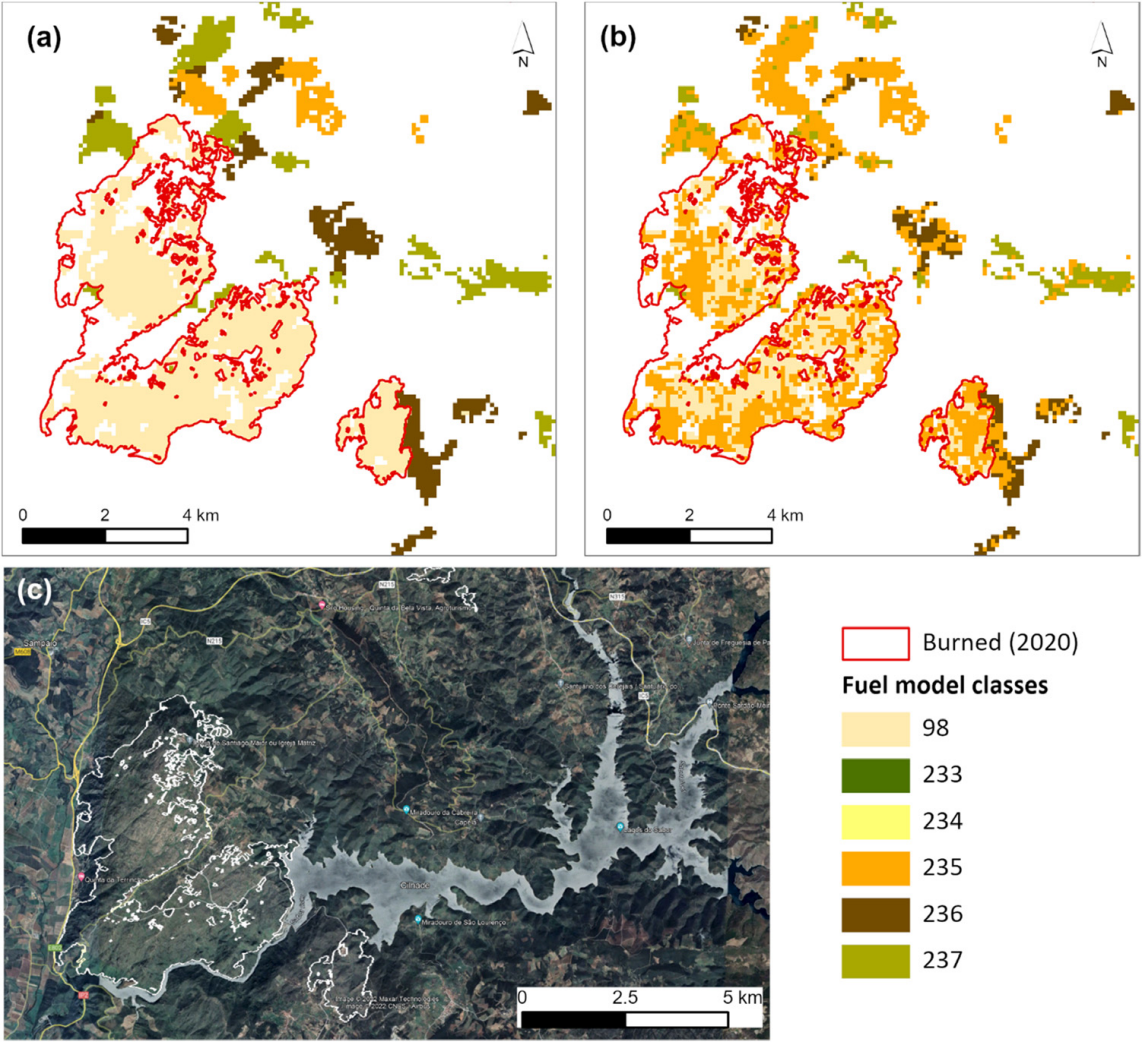
Example of a fire that burned in shrubland (COS2018) in July 2020 in Portugal. The fuel model classes assigned depend on TSLF (shrub age) with rules set from combining authors and fieldwork experience (a); the updated SM5 for this area results from using the rules based on thresholds from the SAVI composite for June of 2021 (see text for details) (b). Different levels of vegetation cover inside the burned area perimeter are shown in the basemap true-color composite (Fig. 6c). Fuel model classes correspond to: 98=unburnable; 233=V-MAa; 234=V-MAb; 235=V-MH; 236=V-MMa; and 237=V-MMb (see Table S1 for descriptions).

Fig. 6 shows the perimeter of a large burned area from July 2020 for the FM map of 2021, assigned to the unburnable class (FM 98) because it has only 1-year-old fuels (Fig. 6a). The fuel model map is updated using SAVI data from June 2020, showing sparsely vegetated areas (FM 235) due to vegetation regrowth after the fire (Fig. 6b).

### SM6: Fuel models in burned eucalypt forest - uses dataset C (fire-atlas) and F (fieldwork)

**Fig. 7:**
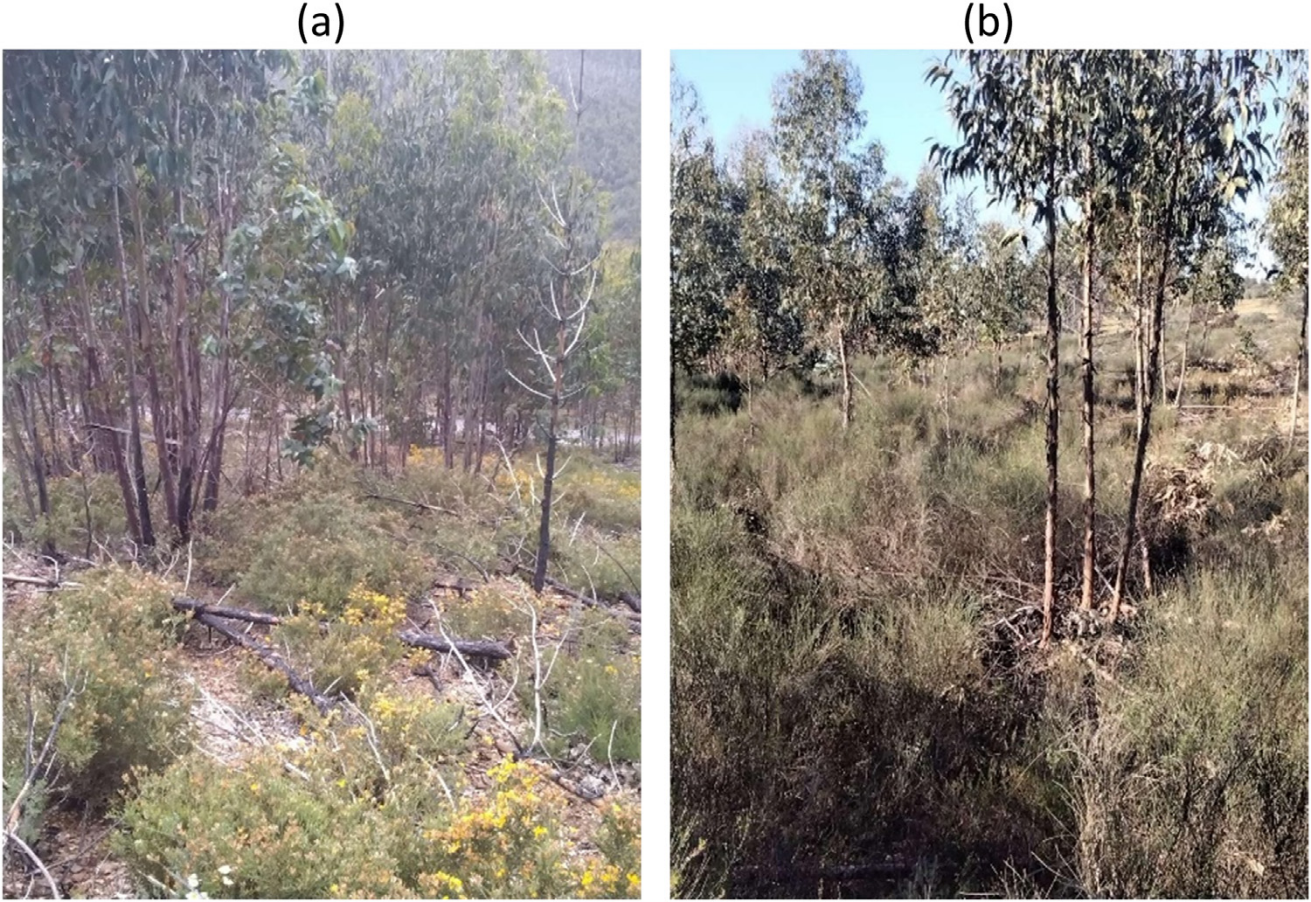
Two examples of surface vegetation in eucalypt plantations five years after the fire. The understory is mainly composed of tall shrubs and woody debris. Photos are from fieldwork done in the Central region of Portugal.

A different fuel model class is assigned to eucalypt forests given the fast-growing characteristics of the species and the prevailing lack of active forest management by non-industrial owners in Portugal. To update fuel models in burned eucalypt areas we considered TSLF, authors experience and fieldwork (Fig. 7) in central Portugal [25]. Regeneration in unmanaged eucalypt plantations is classified as tall shrubs between 3 and 5 years after the fire, since it is the class more likely to represent fuel structure and potential fire behavior in that vegetation type.

### SM7: Fuel models in burned maritime pine forests - uses dataset C (fire-atlas) and F (fieldwork)

Burned areas in pine forests in Portugal have dense regeneration of trees. This is frequently referred to as “dog's hair” stands, because of the highly packed and generally small trees leading to large fuel loads (c.a. 56 t ha^−1^). There is horizontal and vertical continuity of the vegetation, including a frequent component of tall shrubs. These areas are usually prone to extreme fire behaviour. None of the national fuel model classes describes this type of vegetation and the alternative is to assign the NFFL 4 fuel model, which is the most similar in terms of fuel load and depth and fire behaviour. However, where fire frequency is high it can lead to potential regeneration failure [37,38]. Therefore, besides TSLF, mean fire return interval (FRI) was also used in the rules of fuel model class assignment. Authors background as foresters and experience as fire analysts, combined with fieldwork contributed to the definition of the following rules in pine stands regenerated after past fires:

(a) IF 10 < TSLF ≤20 THEN FM=4.
(b) (IF TSLF > 10 years AND FRI < 12 years) THEN FM=233/236 (depending whether shrubs are Atlantic (233) or Mediterranean (236)).
(c) IF TSLF >20 THEN 233/236 (depending whether shrubs are Atlantic (233) or Mediterranean (236)).

### SM8: Pulp paper industry properties - uses dataset C (fire-atlas), F (fieldwork) and G (land cover in industry properties)

The pulp industry's eucalypt plantations are actively managed, with scheduled fuel treatments in each stand rotation to protect their assets, reduce fire risk and increase productivity. Detailed land cover spatial data and scheduled fuel treatments were provided by the major pulp paper industries for the time interval from 2019 to 2022. Four groups of FM class assignments are defined to produce the FM map of paper pulp industrial properties:

(a) Productive eucalypt plantations have three rotations of 12 years each. Plantation age is used to assign a different fuel model class in each rotation. A description of the assignments is available in table A1 from this study [25].
(b) Pulp paper industrial properties with other land cover types are also assigned FM classes based on foresters personal experience and fieldwork.
(c) At the year of FM map production, forest harvesting is integrated and FM 11 from the NFFL fuel model typology is assigned to account for downed and dead woody slash from logging.
(d) Where fuel reduction operations are implemented (e.g. manual, harrowing or chemical interventions), FM is updated to 224 because of its low fuel loading and estimated low fire spread rate. Post-harvest coppicing of the regenerating eucalypt stems leaves slash fuels on the surface that can be best represented by FM 1 (RESE-01) from a set of fuel models developed for central Portugal.

Land cover data and geographical information of industrial pulp paper forests and their fuel management plans are confidential, therefore the SM rules defined in each of the previous groups are not sharable. For this SM only the final industrial fuel models maps are shared in the datasets and models repository link.

### SM9: Current year burnt areas exclusion - uses dataset C (fire-atlas)

Areas burned before the main fire season (usually pastoral burns under mild weather conditions) are "unburnable” patches during the main fire season, and thus are assigned the FM 98.

### SM10: Shrublands (Atlantic *versus* Mediterranean) - uses dataset H (BIOLIT)

**Fig. 8:**
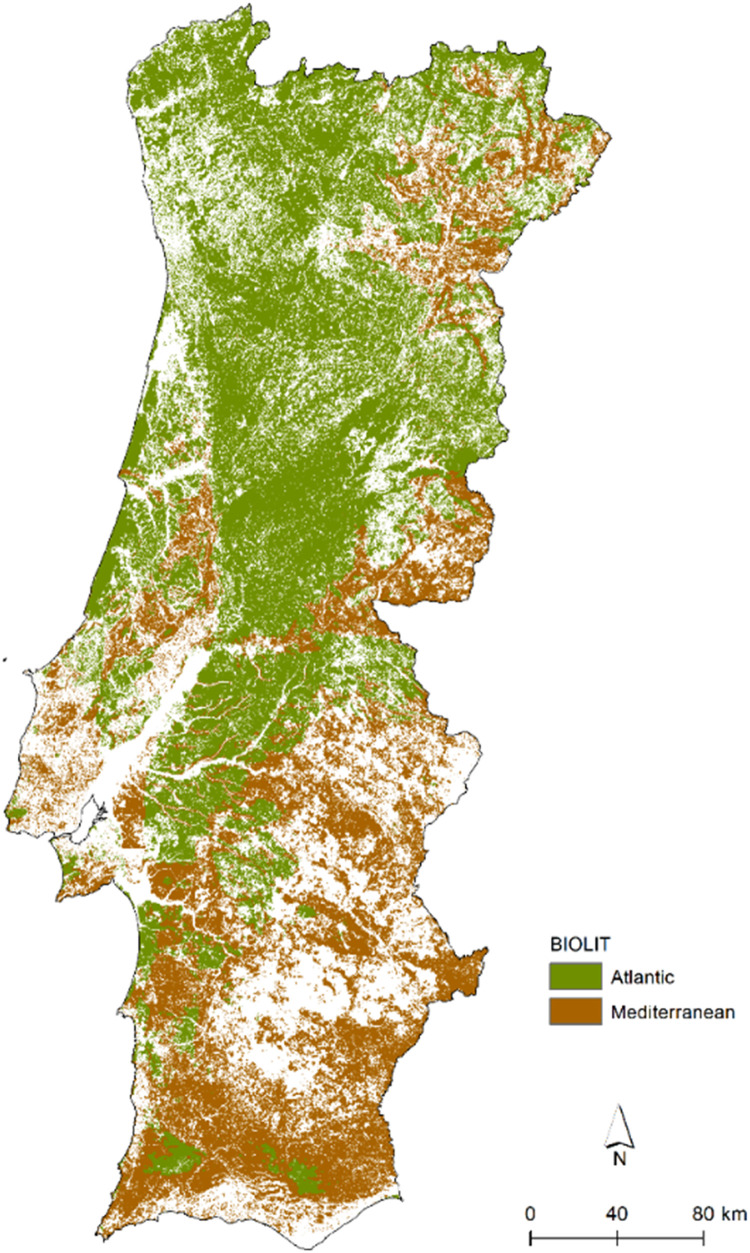
Spatial distribution of the Atlantic *versus* Mediterranean forests and shrublands communities, based on the combination of bioclimatic and lithologic data (BIOLIT). See Fig. S1 for the matrix resultant from all classes combination of both variables.

The Portuguese FM typology distinguishes Mediterranean and Atlantic shrubland communities, as their fuel properties and fire behaviour characteristics are distinct. A potential distribution of these in the mainland of Portugal can be obtained by combining information on the spatial distribution of temperature and precipitation, and the types of soil parent material. The annual ombrothermic index is combined with the soil parent material classification using the matrix shown in Fig. S1. This matrix is based on relationships between plant community types, soil types and climate [39]. For example, forest and shrublands in limestone-derived soils and schist-derived soils under drier climates are classified as Mediterranean communities. The resultant map is shown in Fig. 8. This map is then used to change FM classes in shrublands. For example, if the area is classified as Mediterranean, then shrubs must be assigned the class 236 (V-MMa) or 237 (V-MMb), depending on whether they are tall or low, respectively. In Atlantic areas, shrubs are coded with 233 (V-MAa) or 234 (V-MAb) if they are tall or low, respectively.

### Fuel models maps (FMM)

**Fig. 9:**
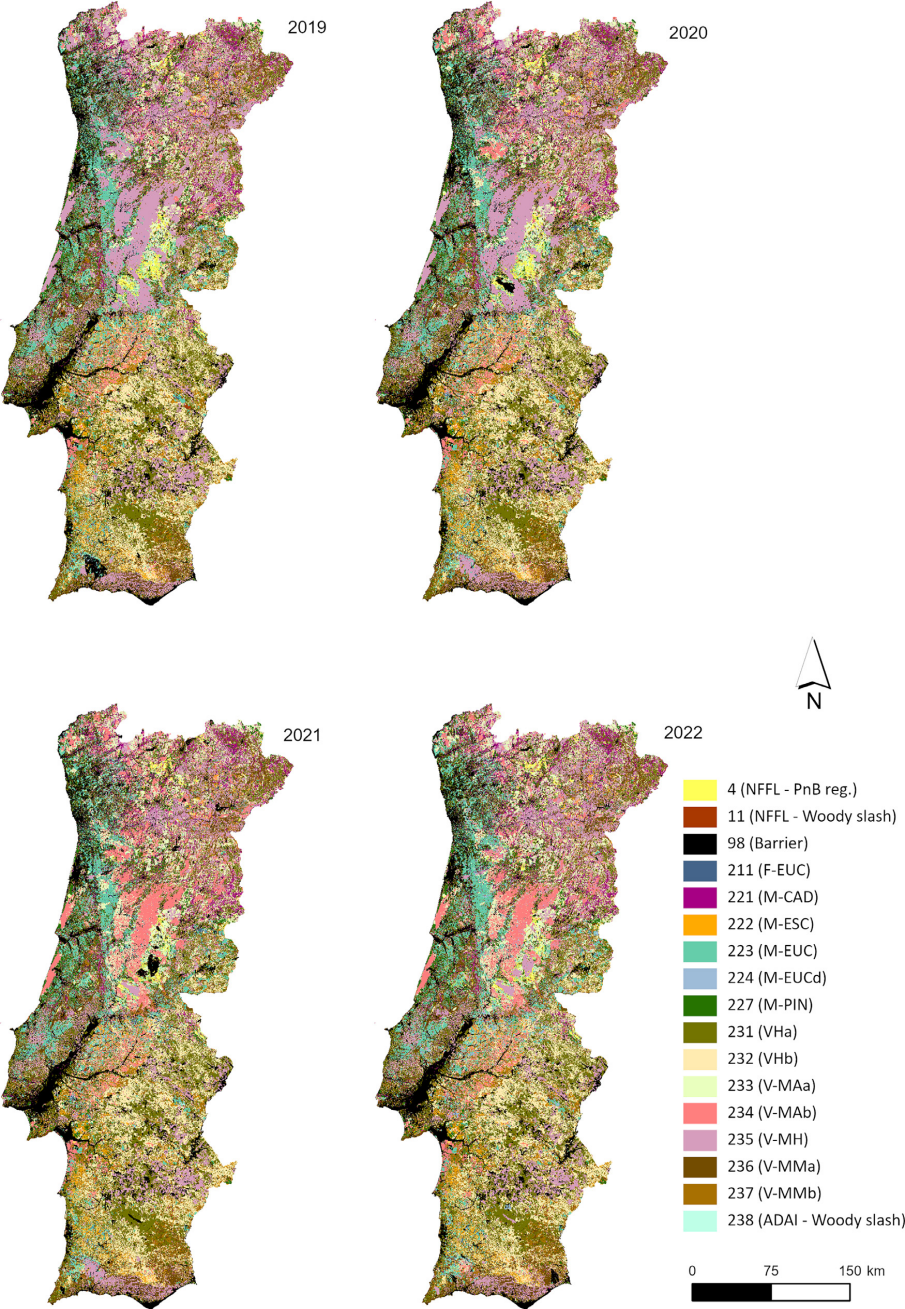
Fuel models maps (FMM) produced after applying the FUMOD model to the national land cover map (2018).

The FUMOD toolbox contains the previously described sub-models and all the datasets compiled in a geodatabase, which are required to obtain FMM from 2019 to 2022. Fig. 9 shows the final maps from running the 10 SM and mosaicking them into each one of the years mapped.

## Assessment of FMM

Fieldwork has an important role in building the rules for fuel models assignment. Because it requires high human resources and is expensive, fieldwork planning was straightforward in either fire-prone areas or land cover types where large variability of vegetation makes fuel model class assignment fraught of uncertainty. Table 4 shows randomly selected areas from the fieldwork, inside the most representative land cover types, to trace back the variables used in the SM to obtain the final FMM from the FMB. A photo for each fuel model classified in the field is shown in Fig. S3.

**Table 4:** Random selection of areas extracted from the fieldwork database to show fuel model assignment in each of the main land cover types in the field. ID=Identifier of the area; LC=land cover; FMB=Fuel Model in the Basemap; TSLF=Time Since Last Fire (DATASET C); FRI=Fire Return Interval (DATASET C); TCD=Tree Cover Density (DATASET D); S2=Sentinel-2 (DATASET E); IP=Industrial Properties (DATASET G); H=Mediterranean/Atlantic classification (DATASET H); FMM= Fuel Model map; Obs_FM=Observed Fuel Model (DATASET F). A photo for each ID is shown in Fig. S3.

**Fig. 10:**
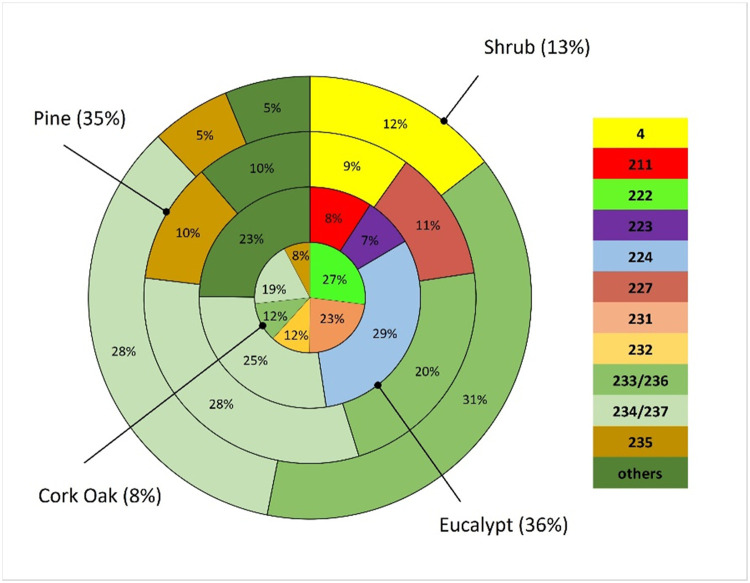
Distribution of fieldwork fuel model classes in each of the main fire-prone land cover types. It is shown four rings, from the inner to the outermost: cork oak, eucalypt, pine forests, and shrublands.

A simple quantitative assessment of the FMM for the years of 2021 and 2022 was performed by comparing them with fieldwork data. The collection of these data were not supported by a statistical sampling design because the areas visited were dependent on the opportunities and resources available for carrying them out. The quantification of intra vegetation type variability is shown in Fig. 10, for pine and eucalypt forests, shrublands and cork oak woodlands, all targeted for fieldwork. Only fuel model classes with frequencies larger than 5 % in each land cover type were selected.

Fig. 10 shows that the variability of fuel model classes is larger in cork oak forests (inner ring) and lower in shrublands (outer ring), which highlights the larger uncertainty in assigning fuel model classes in cork oak forests. These forests are assigned the class M-ESC (222) in the FMB. However, this class only represents 27% of all FM classes observed in this land cover type. The same applies to pine forests, where the fuel model class in the FMB is M-PIN (227) but this fuel model is only present in 11% of the pine forests inventoried. One of the factors related with this variability of surface fuel models classes in pine and cork oak forests may be the influence of tree cover density on the shrub *versus* herbaceous composition and plant species richness in the understory [40]. Almost 50% of pine stands were classified as tall (fuel classes 233/236) or low (fuel classes 234/237) shrublands, frequently in open forest stands.

The next analysis evaluates the potential impact in simulated fire rate of spread and fireline intensity of the misclassification of mapped fuel models. Modelled fire behavior was obtained using the BehavePlus Fire Modeling System [41], under constant midflame wind speed (10 km h^−1^); dead fuel moisture contents of 6% (1-HTR), 7% (10-HTR) and 8% (100-HTR); and live herbaceous and live shrub moistures of 30% and 90%, respectively. These values are commonly used for simulating Portuguese wildfires. Tables 5 and 6 show the percentage of overestimation (green) and underestimation (red) of simulated rate of spread and fireline intensity, respectively, for all the combinations of fuel model classes. We acknowledge that calculations were not done for the litter layer group, since fuel models in this group are not assigned in the FMB (see SM1).

**Table 5:** Crosstabulation matrix that assesses the impact of fuel models misclassification in the simulated rate of spread (ROS). Color scheme ranges from dark red (underestimation) to dark green (overestimation). Interpretation, for example for the largest underestimation (-74 %) is: an incorrect classification of the fuel model class of M-ESC when it should be V-Ha, represents an underestimation of the ROS of 74 %. Columns are the correct fuel model class, while lines are the mapped fuel model class.

**Table 6:** Crosstabulation matrix that assesses the impact of fuel models misclassification in the simulated fireline intensity (FLI). Color scheme ranges from dark red (underestimation) to dark green (overestimation). Interpretation, for example for the largest underestimation (-96 %) is: an incorrect classification of the fuel model class of V-Hb when it should be NFFL 4, represents an underestimation of the FLI of 96 %. Columns are the correct fuel model class, while lines are the mapped fuel model class.

**Fig. 11:**
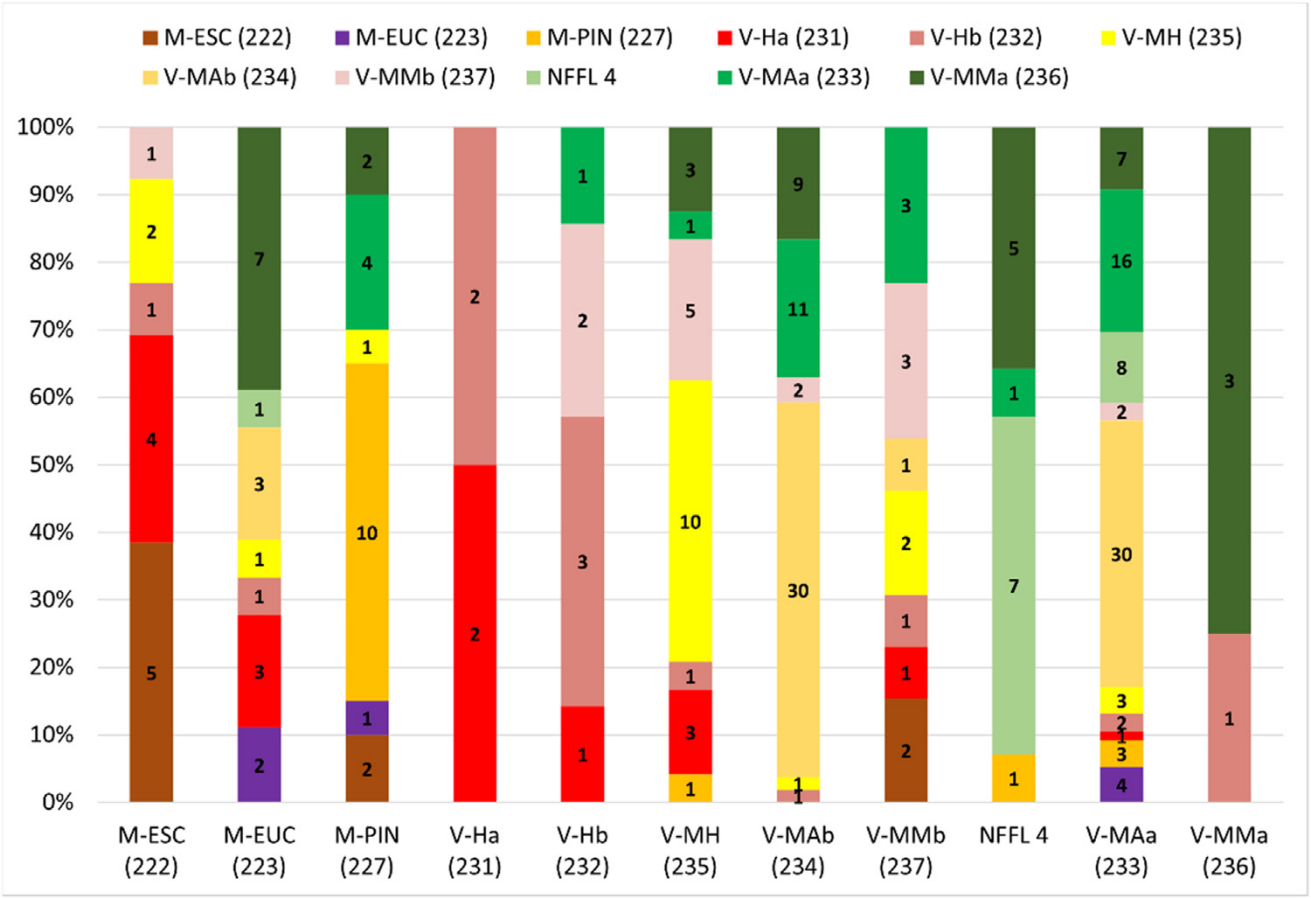
Distribution of fieldwork fuel model classes per fuel model class mapped, in each of the main fire-prone land cover types. Values inside bars represent the number of inventories per fuel model class.

Fig. 11 shows the distribution of fuel model classes observed in the field (Y-axis) in each fuel model class mapped (X-axis), thus providing some information on commission errors. We targeted the analysis in the main land cover types inventoried (Fig. 10).

Combining the information in Fig. 11 with the simulated values in Tables 5 and 6 provides useful information on some of the major limitations in assigning fuel model classes to land cover types. In the case of cork oaklands, the presence of tall grass has a frequency of ca. 30 %, which may represent a 74% underestimation in simulated ROS and a 61% overestimation of FLI. This highlights the importance of improving information regarding the grass layer, especially if estimates of ROS are used to support suppression management operations, where information on the estimated position of the fire front in the next hours is relevant for defining firefighting strategies. In eucalypt forests, almost 40% of the fuel model class is observed in tall Mediterranean shrubs (V-MMa), which does not have a relevant impact in the quality of fire behavior estimates. Pine forests (M-PIN) has ca. 50% of agreement in fuel model class assigned, but 20% misclassifications with fuel model class V-MAa. This error may mainly lead to a 61% underestimation of FLI. This suggests that a better characterization is needed of the main factors leading to an increase in shrubs in the understorey. The intensity of fires in these areas can be high with the corresponding consequences in terms of fire suppression difficulties and fire severity. Regarding grasslands, there is an overall overestimation of ROS resultant from misclassifying them with low grasses (V-Hb) or with low shrubs (V-MMb). In the last, this represents ca. 30% of the cases where the major impact may be in underestimating FLI by 78%. For the sparse vegetation (V-MH), the main commission error is with V-MMb, and may lead to a 34% underestimation of FLI. Both fuel model classes are characterized by low shrubs, and their separability on site based on the degree of coverage is not always easy.

**Fig. 12:**
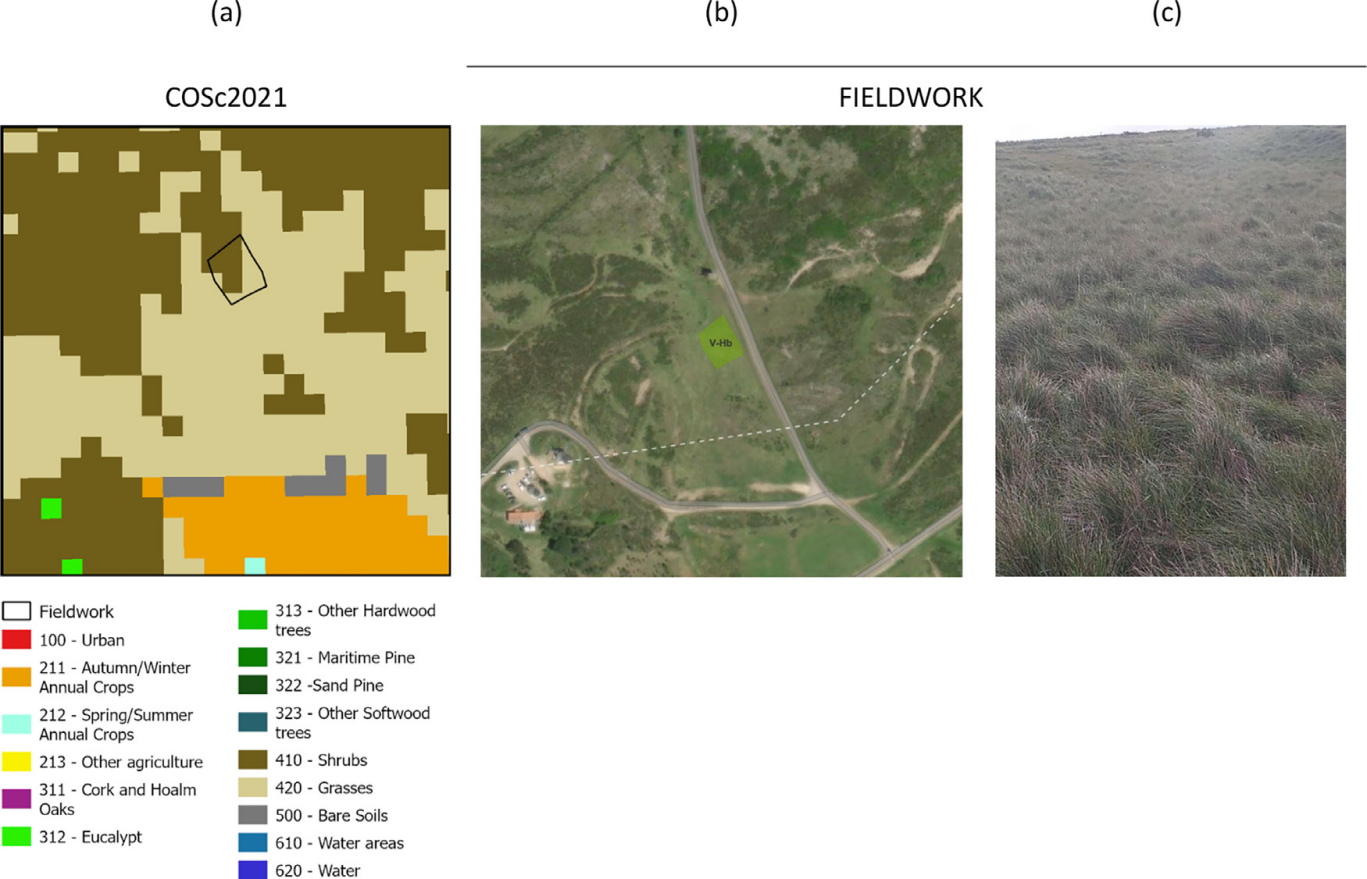
Example of a visited area where land cover type In COS2018 is shrubs (then fuel model assigned in V-MMa (results from SM5), but in the recently developed land cover map of COSc2021 is a mixture of shrubs and grasses (a), which was confirmed in the field (b,c).

The low Atlantic shrubs fuel model (V-MAb) is one of the best classified (ca. 60% of hits) according to fieldwork data. The main commission error is found for the tall shrubs fuel model (V-MAa), which may lead to underestimates of 35% in ROS and 65% in FLI. Distinction between tall and low shrubs was only done for shrublands based on the relationship between the increase in vegetation greenness with TSLF. Research-based information on biomass derived from remote sensing or/and vegetation modelling is needed to better capture fuel accumulation dynamics of shrublands. In Portugal, to the best of our knowledge there is a single study that modelled shrub biomass accumulation with its age [34]. Also, an improved classification of the distribution of Mediterranean *versus* Atlantic shrubs could decrease the variability found in the V-MMb fuel model. In this case, where the true fuel model class is V-MAa (ca. 20%), the underestimation of FLI can be of 79%. In approximately 35% of the sites classified with the NFFL 4 fuel model (with ca. 50% hits), the true fuel model identified in the field is tall Mediterranean shrubs (V-MMb). In this case, both ROS and FLI are overestimated, by 118% and 131% respectively. The same confusion exists in the fuel model class V-MAa, where ca. 40% of the areas are classified in the field as V-MAb, thus as low shrubland. This leads to potential overestimation of fire behavior, especially of FLI (185 %). Finally, V-MMa is the best mapped fuel model, with more than 70% of hits. In this case, there is a single misclassification with V-Hb fuel model. We investigated this (Fig. 12) and according to COS2018 the land cover is shrubs. However, in a recently developed land cover map for Portugal with a spatial resolution of 10 m [42], the occupation is a mixture of shrubs and grasses (Fig 12b), which was confirmed by fieldwork (Fig. 12c). This shows the need to have more recent land cover products to update the vegetation type and improve que quality of final FMM, at least in some classes. Overall, the comparison with fieldwork data highlighted some important limitations of the current FMM datasets that will be discussed in the next section.

## Discussion

We presented FUMOD, a flexible and customized method to map annually mainland Portuguese fuel models, based on featured variables that can contribute to improve the quality of fuel models maps. A model basemap is obtained from assigning fuel model classes to land cover types. Neither in Portugal nor in other countries from Europe is land cover mapping produced annually. Therefore, any other variables derived from the land cover map need to be updated because of several factors affecting vegetation changes. Depending on data availability and quality, the main goal is to better capture vegetation heterogeneity and dynamics (due to disturbances) and provide an updated and reliable fuel map, which may have a relevant impact on the quality of fire simulations. One of the challenges is to have geographical data at the minimum of 100-m spatial resolution. The spatial resolution of the FMM should be based on the best available land cover map resolution. The fuel model classification system adopted should represent the prevailing vegetation type and fuel complex, based on fire spread and behavior properties. Therefore customized fuel models should be developed to support fire spread and behavior simulations. The method proposed can be extended to other Southern European countries using, for example, the next datasets (some of them are used in this work; see Fig. 3):

– Fuel model typologY: the set of 40 standard fire behavior fuel models developed for the US by Scott and Burgan [13] despite not being validated with observed fire behavior data, were obtained from a combination of vegetation physiognomy, climate (arid to semiarid, defined as rainfall-deficient in summer; subhumid to humid, with adequate rainfall in all seasons), fuel loading levels and similarity in simulated fire behavior. They might be considered appropriate for use in fire-prone Mediterranean landscapes, whenever a typology of its own does not exist.
– Dataset A: Copernicus Global Land Cover dataset (to define the FMB map).
– Dataset B: CORINE land Cover map (to mask out irrigated agriculture, for example).
– Dataset C: European forest fires database (EFFIS) with burned area perimeters (from 2000 onward). This is used to calculate TSLF and FRI used in some SM of FUMOD; any other fire perimeters database available at the country level can be used.
– Dataset D: Tree Cover Density from the Copernicus Pan-European High-Resolution Layers. Forest definitions adopted by major international environmental and forestry organizations [43] can be used to set the TCD thresholds rules used in the SM3.
– Dataset E: Sentinel-2 derived spectral vegetation indexes (NDVI and SAVI were derived in this study, but others could be calculated) can be easily calculated for any country. In each case, an analysis of the values in historical burned areas as a function of TSLF is recommended, since the vegetation type, spatial heterogeneity and soil type may change the threshold reflectance values used in SM4 and SM5.
– Dataset F: fieldwork done to assess or validate fuel models, and to guide the definition of user-defined rules built for each SM.
– Dataset H: to the best of our knowledge there isn't any other grid of the spatial distribution of Mediterranean *versus* Atlantic forests and shrublands communities. The matrix that combines bioclimatic and lithologic data (BIOLIT) can be adapted by the user; alternatively, the Koppen-Geiger climate classification can be used [44].
– SM9: if available, other detailed land cover and fuel data can be used (e.g., information on owners forest management operations, such as pruning and thinning or coppicing). Fuel load reduction resulting from mechanical treatments or prescribed burning should be considered given their impact on the fuel model assigned.

The shared FUMOD toolbox is user friendly, easily understood by anyone with geomatic skills. Its flexibility lies in allowing the FMB to be updated with different sources of geographical data to better characterize the current state of vegetation and extant fuel models. This flexibility, aiming at improving FMM quality, at the best spatial resolution, is extremely important to support operational fire management. For example, updated FMM are required as input in fire spread simulations to help fire suppression decisions. However, careful consideration of its use for research purposes is warranted. For example, to calibrate a fire spread simulation system, the impact of each fuel map update (SM) in fire spread and behavior estimates should be evaluated separately. A research question could be: in FUMOD, does DATASET G improves the quality of fire spread estimates? (work in progress in another study developed by the authors). This will provide information on the most important variables to consider, further research needs and can aid in defining fieldwork campaigns. Also, annual fuel models maps produced without a systematic approach (relying on the same variables) may undermine their inter- or intra-annual comparisons. Using recently developed land cover maps for Portugal to derive variables used in any SM or in obtaining the FMB, also limits the comparison with previous years and the approach used in fire spread model calibration.

Nonetheless, additional data and further research can be integrated in future developments of FUMOD toolbox, to improve the quality of final FMM, thus of fire spread and behavior estimates, namely:

– It is important to investigate the contribution of agricultural fires for the whole country total burned area. With some inter-annual variability, agricultural fires represent c.a 10% of the total annual burned area extent in Portugal and there are no fuel models developed for this land cover type. Depending on the agricultural class, fuel model assignment was based on the expected fire behavior, more similar in some agriculture classes to the presence of a grass layer, or a combination of this with low shrubs depending on the presence or not of natural vegetation;
– It may be relevant to investigate how likely different agricultural classes burn with distance to urban areas; some agricultural classes may not burn within a distance to urban areas (for example, due to active fire suppression or type of agriculture), and this likelihood can decrease significantly with distance; this may lead to different fuel model assignments as distance to urban areas increase;
– Some of the areas classified as urban areas affected by fire more often than desired. Rural communities are frequently embedded within a vegetation matrix, including a surface-overstory fuel continuum (for example due to agricultural abandonment). To better describe this situation, built-up areas can be assigned non-burnable only where its structure density is high. The European Settlement Map from 2015 or the more recent Imperviousness Hight Resolution Layer [45] could be used to mask out those areas and assign a low fire spread fuel model for the remaining area;
– S2-derived fuel moisture index or single bands (e.g. NDMI - Normalized Difference Moisture Index [46]) can be used to mask out areas that are unlikely to burn due to their high vegetation water content;
– A new annual land cover map (COSc) for mainland Portugal was released for the years of 2018, 2020, 2021, and 2022 with only 15 classes but 10 m of spatial resolution. This map was derived from automatic classification of S2 imagery, using artificial intelligence and machine learning algorithms [42]. This product can be used to decrease the uncertainty in fuel model class assignment in open forests (e.g. oaklands, Fig. 10), a class that does not exists in COS2018. In DATASET A (COS2018), the forest class does not have different tree cover fractions, thus fuel model class assigned is that of a mixture of litter and shrub surface fuel layers. In the higher spatial resolution of COSc, a forest patch in COS2018 can have a mixture of trees, shrubs and grasses. However, the lower thematic resolution of the former is a disadvantage in the FMB classification.

In this work we assessed the quality of produced FMM without validating them. The use of fieldwork data for validation of FFM maps should be based on a statistical sampling design to produce reliable accuracy metrics. One approach could be to define the intensity of inventories based on fire incidence in each vegetation type and dependent on the number of potential fuel model classes that can be assigned to each one. Statistically, this would require a large sample size, also dependent on the number of variables considered to relate with the distribution of vegetation and fuel types in Portugal. A sampling strategy could be set by selecting only the most frequent fire-prone vegetation types and fuel model classes, and by adding several inventory crews. However, the latter will increase costs extremely. At least in Portugal, because fieldwork is extremely time consuming and expensive, funds directed to the study of vegetation or specific projects are needed to cover costs and follow an appropriate sampling design.

In the previous section we compared FMM with fieldwork done in 2021 and 2022, using a Survey 123 ArcGIS app developed for that purpose. One major advantage of this application is its simplicity and speed in the collection of the fuel model class. Data are collected systematically and stored in a geodatabase that can be easily processed in a GIS program. It contributes to detect errors in fuel model classification, improve user knowledge, define model's rules, and the quality of the updated fuel model map. Photos are included in the geodatabase and are essential to observe intra-vegetation patch heterogeneity. This application can also be used in other fuel-model related studies, for example, to monitor forest and fuel management activities, or vegetation recovery after a fire or other natural disturbances that affect fuel loading and patterns. Now the manual of the application is in Portuguese because it was developed for its use in Portugal by foresters and fire personnel that may have difficulties with a non-mother language. However, soon it will be translated to English for its use by researchers from other Mediterranean countries, who may want to use the Portuguese fuel models typology in their fire management. Nonetheless, a similar fuel models survey can be developed for other fuel model typologies using the simple classification approach shown in Fig. S2.

The comparison between the two FMM with the field data points to future work fostering the improvement of the quality of the updated FMM, either through further research and by integrating new variables able to better capture land cover type and fuel build-up dynamics. Two main considerations are:

– Some land cover types have large internal variability of surface fuels, either because they have some source of active management (for example, eucalypt forests), they are agroforestry systems, or they are open forests. The higher spatial resolution Portuguese land-cover map (COSc) can provide detailed information on the spatial distribution of the grass and shrubs layer in the understorey; and
– Further research is required to characterize the distribution of tall *versus* low shrubs. This information may be derived using shrub biomass modelling (always including the variable time since disturbance), which should also include fieldwork for model validation; and/or use of LiDAR data to characterize the structural properties of surface fuels. Satellite data can be used to produce information of productivity of open shrublands communities, which can be used in modelling to estimate fuel loadings and relate them with the adequate fuel models.

The first European fuel cartography (for 2000) was produced by the European Forest Fire Information System (EFFIS, 2017) by using land cover and vegetation maps, and the NFFL fuel models system. There are limitations to produce a wide European common fuel model system since fuel models are site specific and should be applied in the regions for which they were developed to produce more realistic fuel mapping [47]. More recently, a hierarchical fuel classification system consisting of 20 fuel types (both surface and canopy) was developed at the European level, based on land cover, biogeographic datasets and bioclimate modelling [48]. However, one limitations of this fuel map is its spatial resolution of 1-km, which may undermine its accuracy and the quality of fire spread and behavior simulations. At 1-km grid cell size, the within pixel variability is likely high, as the uncertainty in fuel model assignment, both increasing in typical heterogeneous landscape from Mediterranean regions. Because fuel model identification is affected by several sources of error, for example the generalization of fire behavior (with different user-dependent perceptions) in a fuel complex, and the error due to some non-fuel fire behavior factors, it may be acceptable to produce fuel model maps at a spatial resolution larger than 100 m. Here the main limitation is the spatial scale of the land cover map used. A challenging future work can be to expand our method to Southern Europe, with an unique fuel model system classification or different maps resulting from customized nationwide fuel models. We showed that, excluding the industrial private data, all the remaining datasets to produce an updated fuel models classification map can be produced at 100-m cell size.

## Conclusions

The proposed method automatically outputs FM maps from the Portuguese land cover types since 2019, afterwards integrating available sources of spatial data on vegetation and historical burned areas, to have updated fuel models at the beginning of each main fire season. It results from combining expert knowledge (in a rule-based definition) with several sources of data and fieldwork, to obtain a final FM map. The main objective of these updates is to integrate the dynamics of vegetation due to its removal by fires, forest logging and, when available, changes in surface fuels due to private forest management operations. Summarizing, the developed model integrates: 1) the masking out of permanently irrigated agriculture; 2) FM assigned differently in burned areas, and in shrublands in particular, depending on time since last fire; 3) changes in FM classification according to tree canopy cover in some land cover types; and 4) detailed land cover data from main pulp industry companies. Furthermore, the method flexibility allows inclusion or exclusion of SM, according to the national availability and quality of data. The FUMOD will be continually updated and improved as more information and research is integrated.

The full model and its sub-models are available as supplementary files and they can be adapted considering similar objectives. Agricultural areas need further research to adapt assigned fuel models. Also, a deeper analysis is needed to better characterize the relationship between tree cover density and understory layers. Shrubland structure and productivity information needs to be investigated using modelling and remote sensing tools. This method can be implemented in any southern European country, considering the quality of data available.

## Related research article

Sá, A.C.L.; Aparicio, B.A.; Benali, A.; Bruni, C.; Salis, M.; Silva, F.; Marta-Almeida, M.; Pereira, S.; Rocha, A.; Pereira, J.M.C. Coupling wildfire spread simulations and connectivity analysis for hazard assessment: A case study in Serra da Cabreira, Portugal. Nat. Hazards Earth Syst. Sci. Discuss. 2022, 2022, 1-35.

## CRediT authorship contribution statement

**A.C.L. Sá:** Conceptualization, Methodology, Writing – original draft, Writing – review & editing. **A. Benali:** Methodology, Writing – review & editing. **B.A. Aparicio:** Methodology, Writing – review & editing. **C. Bruni:** Methodology, Writing – review & editing. **C. Mota:** Software. **J.M.C. Pereira:** Methodology, Writing – review & editing. **P.M. Fernandes:** Methodology, Writing – review & editing, Data curation, Supervision.

## Declaration of Competing Interest

The authors declare that they have no known competing financial interests or personal relationships that could have appeared to influence the work reported in this paper.

## Data Availability

A link is shared to a repository where the data and models are shared.

## Appendix Supplementary materials

Image, application 1
