# Supplementary material for: A method to produce a flexible and customized fuel models dataset
Source: MethodsX. 2023 May 19;10:102218. doi: 10.1016/j.mex.2023.102218 (PMC10244702; doi:10.1016/j.mex.2023.102218)
Supplement: Supplementary file 1 [file mmc1.docx]

**Table S1.** Short description of the Portuguese fuel models classes (Fernandes et al., 2009) and a corresponding example picture. Fuel models are organized in three groups: **F** (litter); **M** (mixed: litter and understorey vegetation); and **V** (understorey vegetation, shrubland or grassland).

| **Group** | **#** | **Code** | **DeSCRIPTION** | **EXAMPLES** |  |
| --- | --- | --- | --- | --- | --- |
| F (Litter) - The fire behaviour is driven by the litter layer | **211** | F-EUC | Eucalypt litter | Eucalypt plantations |  |
|  | **212** | F-FOL | Litter of deciduous or evergreen hardwoods (2-5 t/ha) | Deciduous oak forests, chestnut, birch and beech. Dense sclerophyllous forest, namely of cork oak and holm oak |  |
|  | **213** | F-PIN | Medium- to long-needle conifer litter (4-7 t/ha) | Pine forests of *P. pinaster, P. pinea, P. halepensis, P. radiata* |  |
|  | **214** | F-RAC | Short-needle conifer litter. The amount of woody debris can be substantial (4-6 t/ha) | Forest stands of *Pseudotsuga*, *Cedrus*, *Cupressus*, *Chamaecyparis*, *Pinus sylvestris*. Other highly-packed litters cab be included, e.g. *Acacia* spp. |  |

| M (mixed) - Both litter and understorey vegetation have significant coverage and drive the spread of fire | **221** | M-CAD | Deciduous litter and a shrub understory, usually with plenty of live fuel (8-17 t/ha) | Oak, chestnut, birch, and beech stands |  |
| --- | --- | --- | --- | --- | --- |
|  | **222** | M-ESC | Evergreen sclerophyllous hardwood litter and a shrub understory (7-17 t/ha) | Cork oak and holm oak forest stands |  |
|  | **223** | M-EUC | Eucalypt litter and a shrub understory (9-18 t/ha) | Eucalypt forest plantations |  |

|  | **224** | M-EUCd | Discontinuous eucalypt litter with or without a shrub component (1-4 t/ha) | Young or recently harrowed eucalypt plantations |  |
| --- | --- | --- | --- | --- | --- |
|  | **225** | M-F | Litter and a fern understory | Forest stands, regardless of species |  |
|  | **226** | M-H | Litter and a grass understory (2-5 t/ha) | Forest stands, regardless of species |  |

|  | **227** | M-PIN | Litter from medium- to long-needle conifers with a shrub understory (8-18 t/ha) | Forest stands of *P. pinaster, P. pinea, P. halepensis, P. radiata, P. nigra* |  |
| --- | --- | --- | --- | --- | --- |

| V (understorey, shrubland or grassland) - Fire behaviour is controlled by shrubs or woody vegetation in the shrublands or grasses | **231** | V-Ha | Tall grassland (>0.5 m) (2-4 t/ha) | Lawns, meadows, pastures, recent fallow land. Cereal croplands. Reeds. Agroforestry systems. |  |
| --- | --- | --- | --- | --- | --- |
|  | **232** | V-Hb | Low grassland (<0,5 m) (~1 t/ha) | Lawns, meadows, pastures, recent fallow land. Cereal stubbles. Agroforestry systems. |  |
|  | **233** | V-MAa | Tall shrubland (>1 m) with substantial dead and/or fine foliage (12-27 t/ha) | Heaths, gorses, old broom shrublands; young acacia stands. Open or young forest stands, regardless of the species, with a shrub layer made up of those species. Dense natural regeneration of pines. |  |

|  | **234** | V-MAb | Low shrubland (<1 m) with substantial dead and/or fine foliage (7-14 t/ha) | Heaths, gorses, brooms, junipers shrublands. Open or young forest stands, regardless of species, with a shrub layer made up of those species |  |
| --- | --- | --- | --- | --- | --- |
|  | **235** | V-MH | Low (< 1 m) and green shrubs, often discontinuous and with grass | Shrubland up to 3 years since the last fire |  |
|  | **236** | V-MMa | Tall shrubland (>1 m) poor in dead fuel and/or with relatively coarse foliage (10-19 t/ha) | Shrublands of broom, *Cistus* spp., *Quercus coccifera* and other sclerophyllous Mediterranean species. Young Hakea stands. Brambles. Open or young forest stands, regardless of species, with a shrub layer made up of those species |  |

|  | **237** | V-MMb | Low shrubland (<1 m) with little dead fuel and/or with relatively coarse foliage (4-8 t/ha) | Shrublands of broom, *Cistus* spp., *Quercus coccifera* and other sclerophyllous Mediterranean species. Young Hakea stands. Brambles. Open or young forest stands, regardless of species, with a shrub layer made up of those species |  |
| --- | --- | --- | --- | --- | --- |

Fernandes, P., Gonçalves, H., Loureiro, C., Fernandes, M., Costa, T., Cruz, M. and Botelho, H. (2009). Modelos de combustível florestal para Portugal, in Actas do 6o Congresso Florestal Nacional. Sociedade Portuguesa de Ciências Florestais; SPCF: Lisboa, Portugal, 2009. [online] Available from: https://www.researchgate.net/profile/Paulo-Fernandes-6/publication/261708410_Modelos_de_Combustivel_Florestal_para_Portugal/links/00b7d53524bec08267000000/Modelos-de-Combustivel-Florestal-para-Portugal.pdf (Accessed 23 August 2021).

The NFFL American fuel models (Anderson, 1982) were used to represent two distinct vegetation types not included in the Portuguese fuel models.

| NFFL Fuel models | **4** | NFFL 4 | Very dense regeneration of maritime pine with a height of 3-6 m (~32 t/ha), typically 5-15 years old | 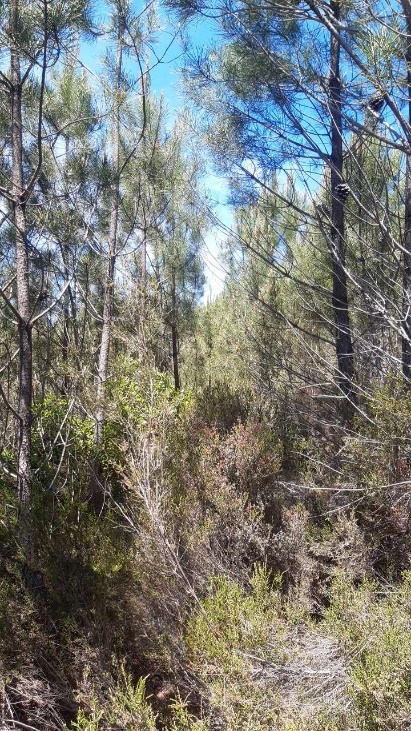 |
| --- | --- | --- | --- | --- |
|  | **11** | NFFL 11 | Downed and dead woody slash from logging, often interspersed with herbaceous vegetation (~28 ton/ha). Forest stands/plantations after harvest or heavy pruning and thinning or coppicing. | 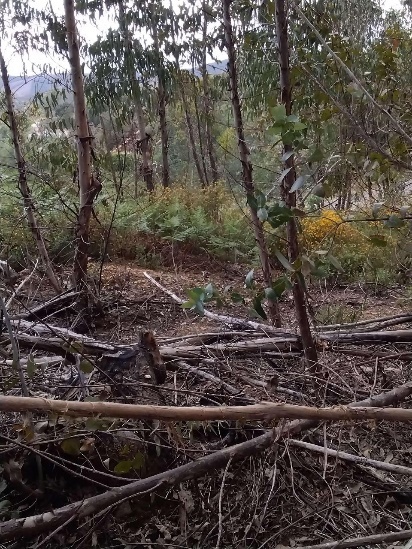 |

Anderson, H. E.: Aids to determining fuel models for estimating fire behavior, USDA Forest Service, Intermountain Forest and Range Experiment Station. [online] Available from: https://www.nwcg.gov/sites/default/files/training/docs/s-290-usfs-aids-to-determining-fuel-models.pdf, 1982.

Another National Fuel Models classification (Cruz, 2005) was used to represent other exploitation wood residuals, with lower fuel load than the one from NFFL, as is for example the case of selection of poles in eucalypt plantations.

| ADAI Fuel models | RESE-01  (238) | Downed and dead woody slash from the selection of poles in eucalypt forest plantations | 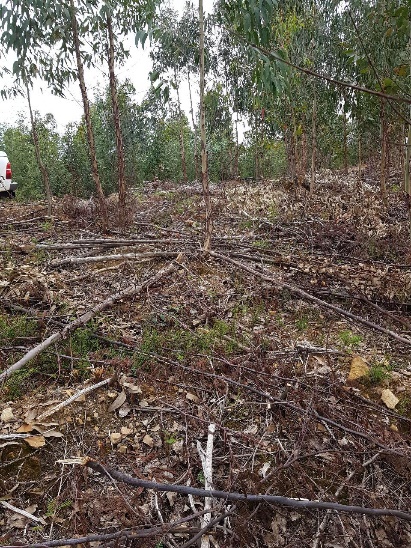 |
| --- | --- | --- | --- |

Cruz, M. G.: Guia fotográfico para identificação de combustíveis florestais – Região Centro de Portugal, Coimbra, Portugal, 38p, 2005.

**Table S2.** Parameters for the Portuguese fuel models classification system. FL = Fuel Load; SVR = Surface to Volume Ratio; HC = Heat Content; Hx = 1hr fuel moisture extinction content.

| Fuel Model | Thickness | FL (t ha^-1^) | | | | | SVR (m^-1^) | | | HC | Hx |
| --- | --- | --- | --- | --- | --- | --- | --- | --- | --- | --- | --- |
|  | (m) | 1hr | 10hr | 100hr | shrubs | grasses | 1hr | grasses | shrubs | (kJ kg^-1^) | (%) |
| F-RAC | 0,05 | 3,75 | 2,00 | 1,00 | 1,18 | 0,00 | 6500 | - | 4500 | 20500 | 28 |
| F-FOL | 0,15 | 2,67 | 1,27 | 0,69 | 1,16 | 0,00 | 4500 | - | 5000 | 20500 | 25 |
| F-PIN | 0,10 | 6,50 | 1,50 | 0,00 | 0,00 | 0,00 | 5500 | - | - | 20500 | 45 |
| F-EUC | 0,32 | 4,63 | 2,96 | 1,27 | 1,12 | 0,00 | 4200 | - | 5000 | 21000 | 26 |
| M-CAD | 0,63 | 4,54 | 1,87 | 0,61 | 9,08 | 0,00 | 6000 | - | 5000 | 20000 | 30 |
| M-ESC | 0,50 | 5,65 | 1,50 | 0,48 | 7,89 | 0,00 | 5000 | - | 5500 | 20500 | 25 |
| M-PIN | 0,50 | 7,21 | 3,00 | 0,00 | 6,89 | 0,00 | 5500 | - | 6000 | 21000 | 40 |
| M-EUC | 0,64 | 8,37 | 3,81 | 0,00 | 4,51 | 0,00 | 4700 | - | 5000 | 21000 | 32 |
| M-EUCd | 0,40 | 1,37 | 2,89 | 1,59 | 1,84 | 0,00 | 4500 | - | 5000 | 21000 | 26 |
| M-H | 0,10 | 2,71 | 1,00 | 0,00 | 0,10 | 0,66 | 5500 | 8000 | 4500 | 20500 | 30 |
| M-F | 0,30 | 4,50 | 1,50 | 0,50 | 0,48 | 2,35 | 6000 | 8000 | 4500 | 19500 | 35 |
| V-MAb | 0,50 | 6,00 | 0,50 | 0,00 | 7,50 | 0,00 | 4500 | - | 4500 | 21000 | 35 |
| V-MAa | 1,05 | 9,50 | 2,50 | 0,00 | 14,50 | 0,00 | 3500 | - | 4000 | 21000 | 35 |
| V-MMb | 0,90 | 4,00 | 0,50 | 0,00 | 7,00 | 0,00 | 3000 | - | 3000 | 20500 | 20 |
| V-MMa | 1,70 | 6,00 | 4,00 | 0,00 | 13,00 | 0,00 | 2500 | - | 3000 | 20500 | 25 |
| V-MH | 0,55 | 1,00 | 1,00 | 0,00 | 5,50 | 1,50 | 4500 | 8500 | 4000 | 19500 | 25 |
| V-Hb | 0,35 | 0,30 | 0,00 | 0,00 | 0,00 | 1,20 | 6000 | 6000 | - | 19000 | 24 |
| V-Ha | 0,60 | 0,65 | 0,15 | 0,00 | 0,40 | 2,35 | 4000 | 5500 | 4000 | 19000 | 24 |

**Figure S1**. Spatial distribution in mainland Portugal of the main soil parent material types (a); annual ombrothermic index (b); and pairwise combinations of the different classes of the two datasets (bottom panel). Figures in the left and right panels are from the reference works presented in the text.

| (a) | (b) |
| --- | --- |
| 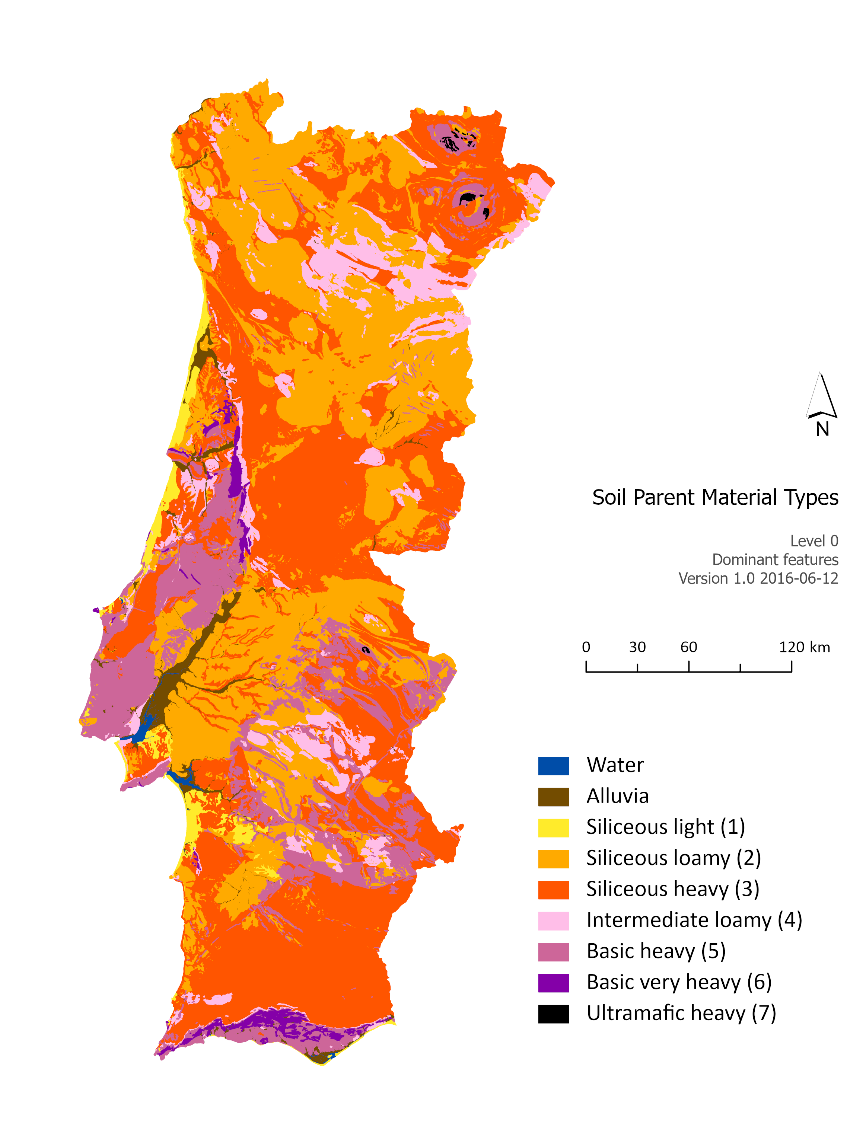 | 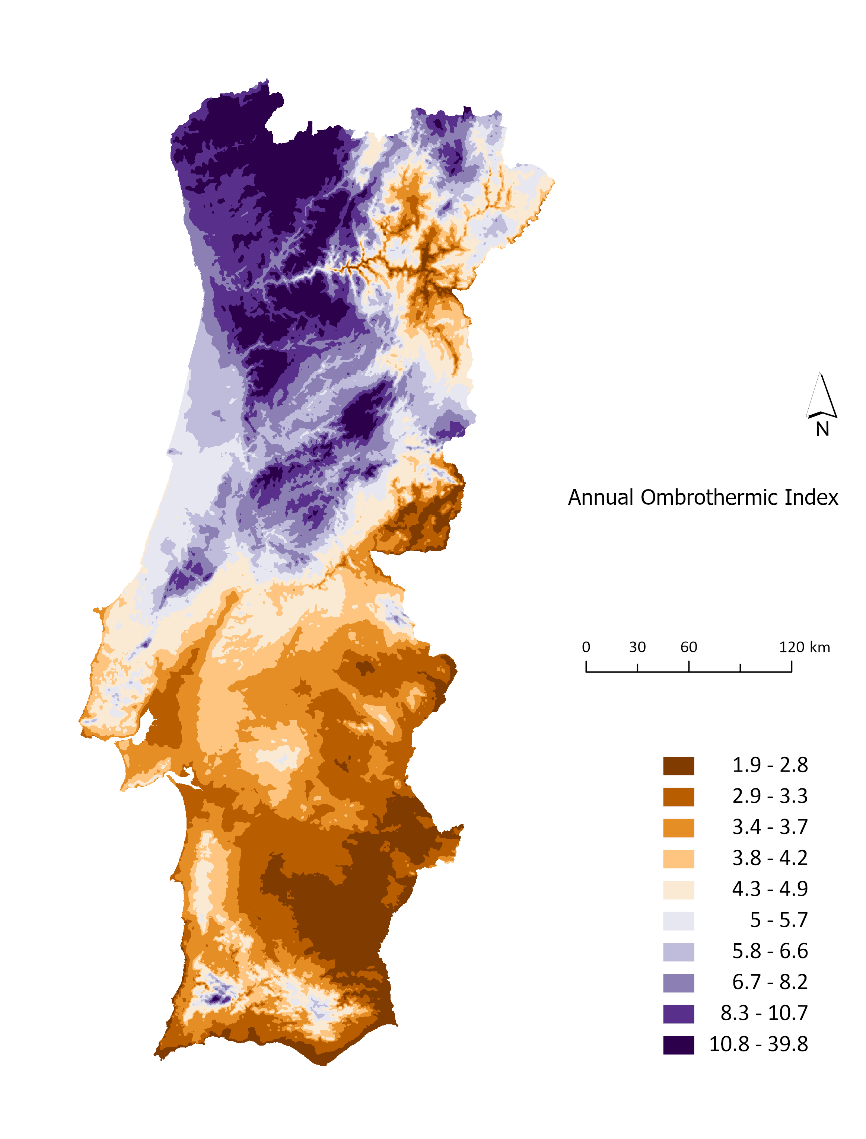 |
|  | |

**Figure S2**. Sequence of steps and IF-THEN-ELSE questions used in the ArcGIS Survey 123 fuel models application.

| (a) | 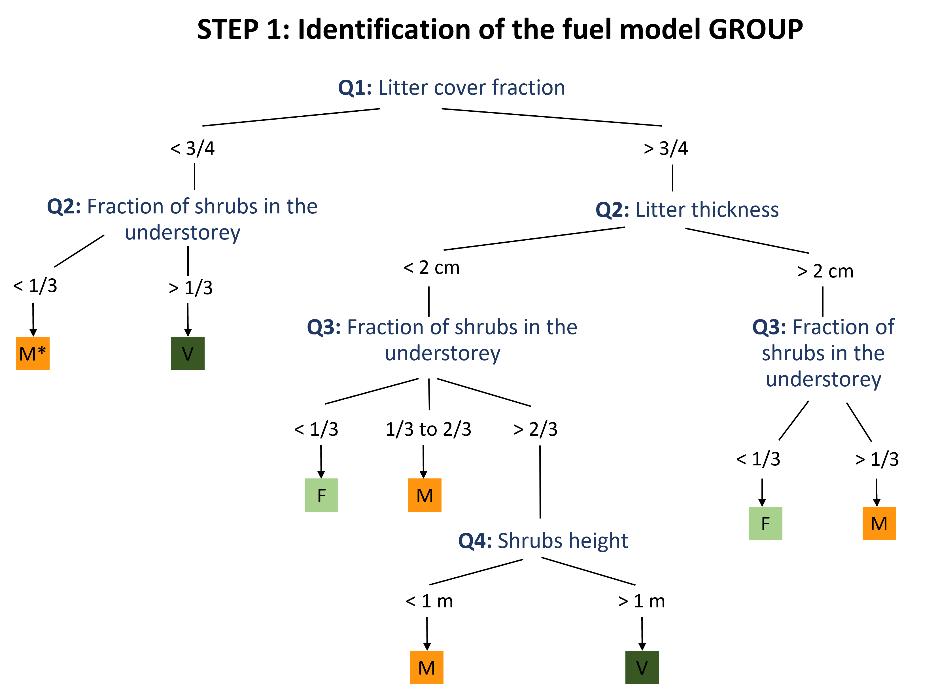 | (b) | 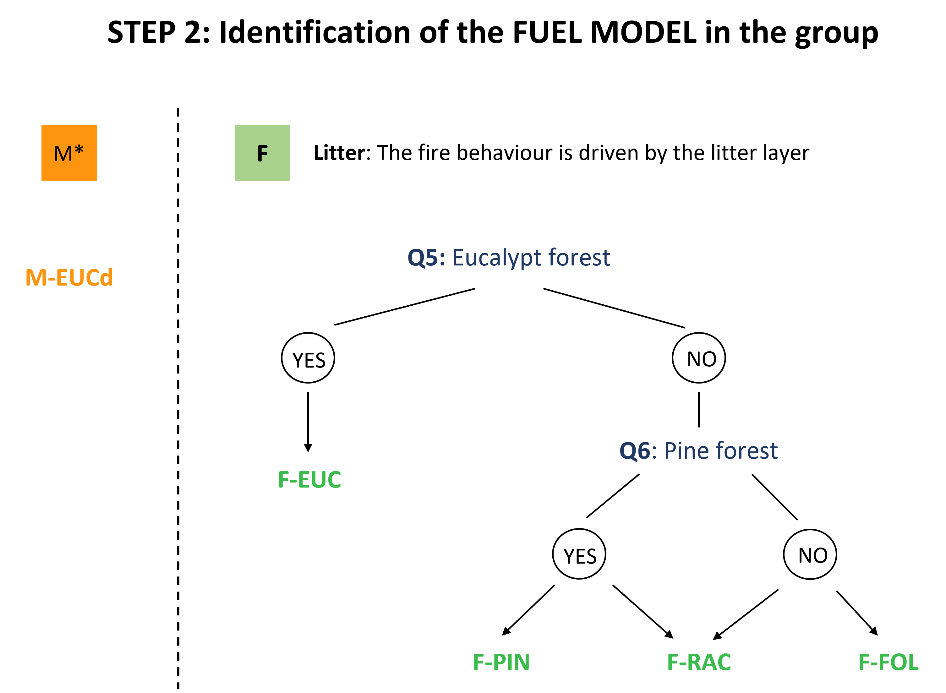 |
| --- | --- | --- | --- |
| (c) | 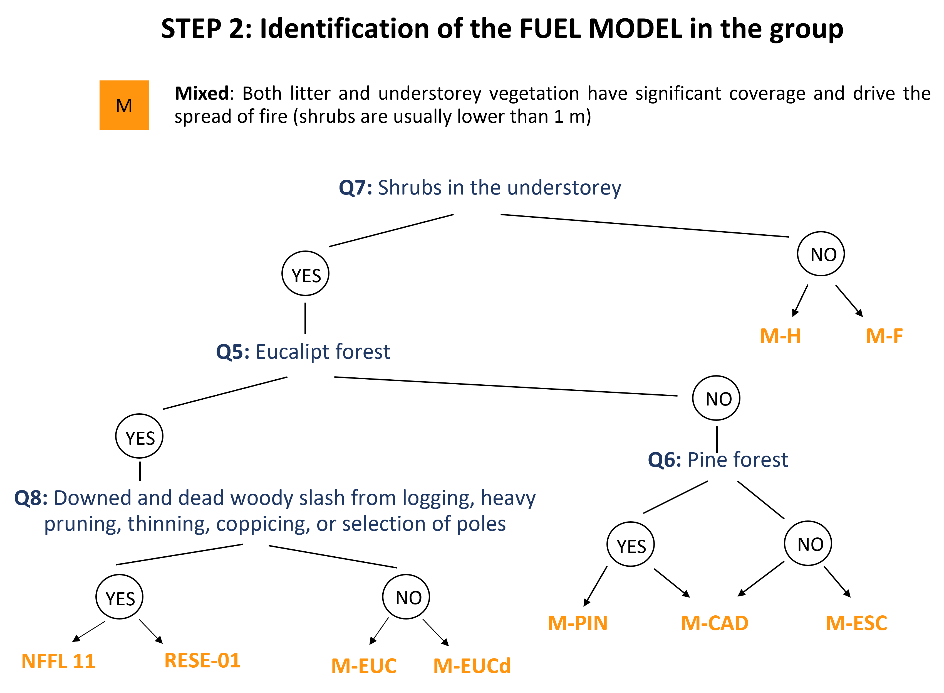 | (d) | 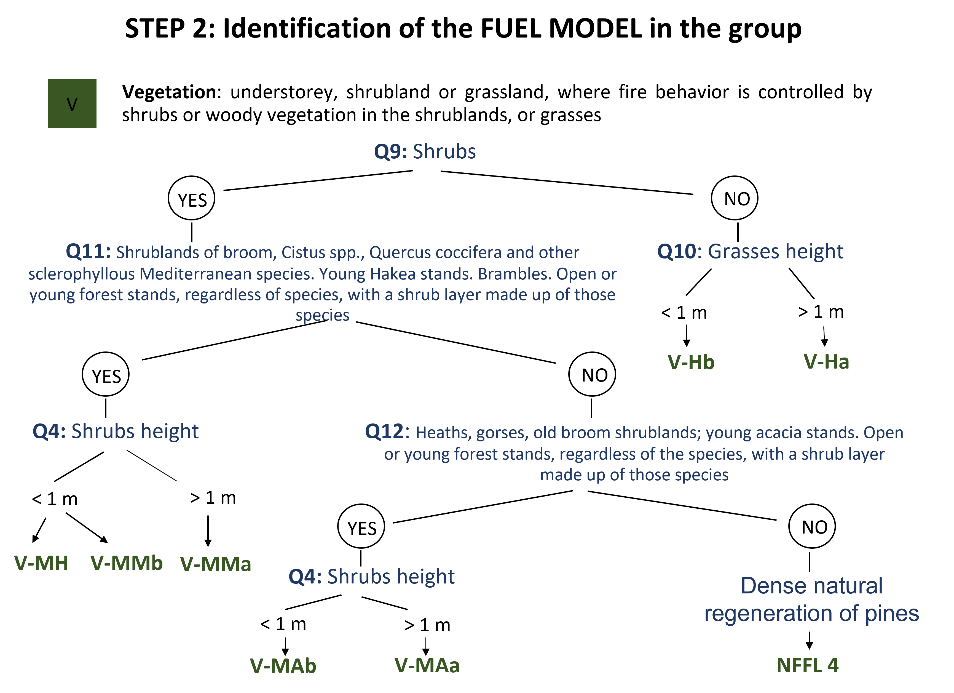 |

**Figure. S3**. Illustrative photos of land cover types and surface fuels for each one of the examples shown in Table 4. These were extracted from the fieldwork database, from selected fire-prone land cover classes (Fig. 2b). IDs are shown in the right corner white box, and have a correspondence with the IDs in Table 4.

| 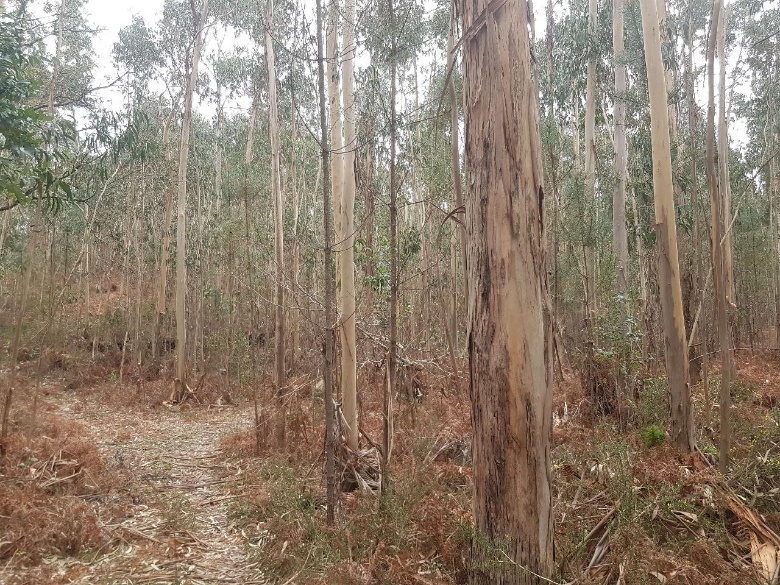  **1** | | 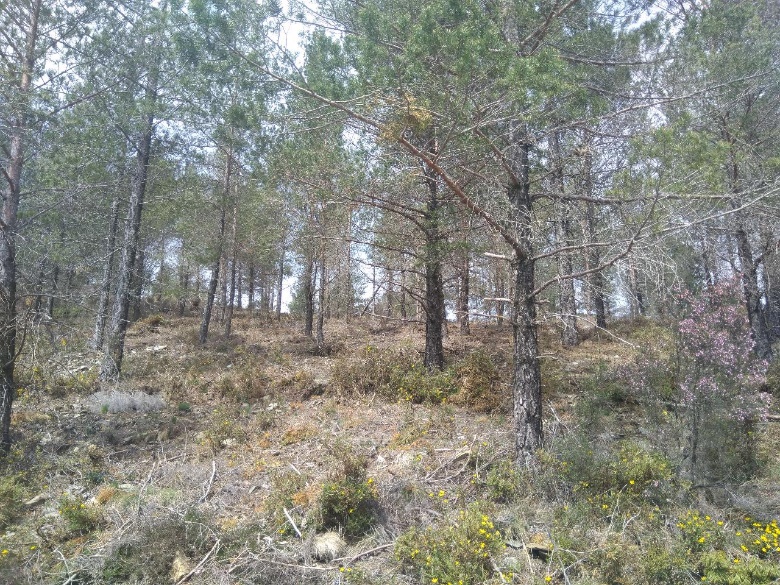  **36** | |
| --- | --- | --- | --- |
|  | |  | |
| 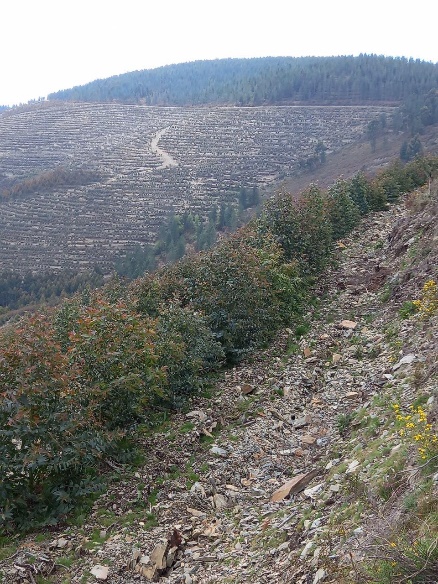  **39** | | 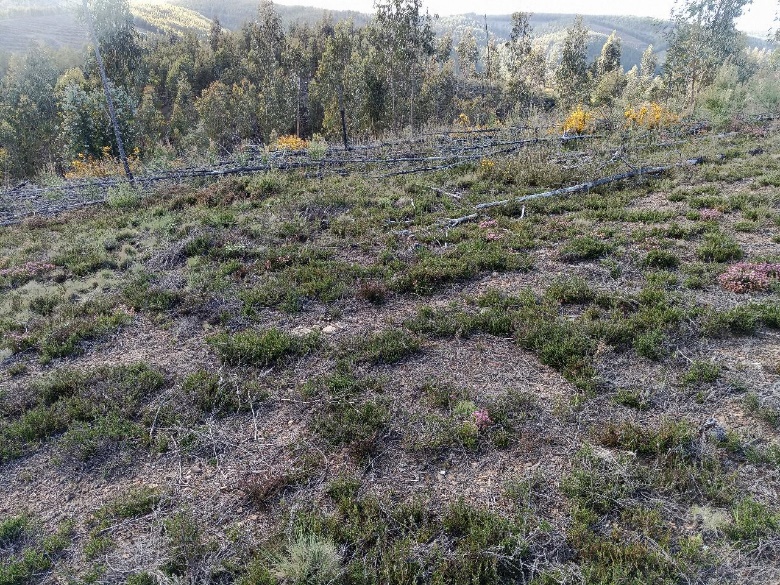  **60** | |
|  | |  | |
| 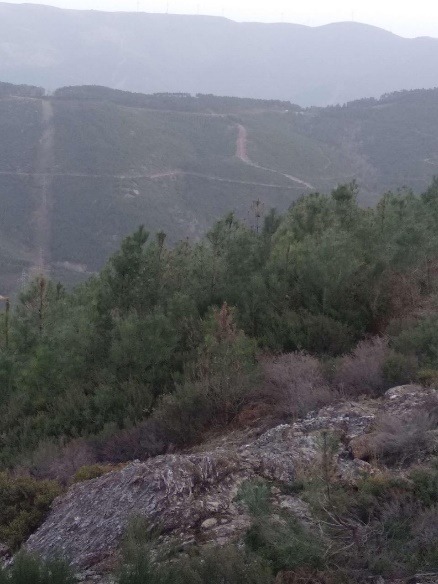  **19** | 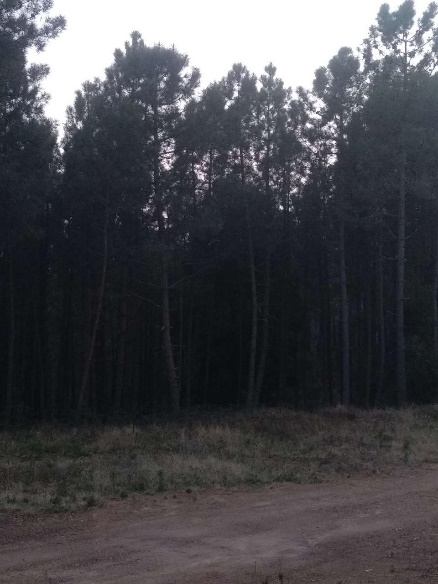  **21** | | 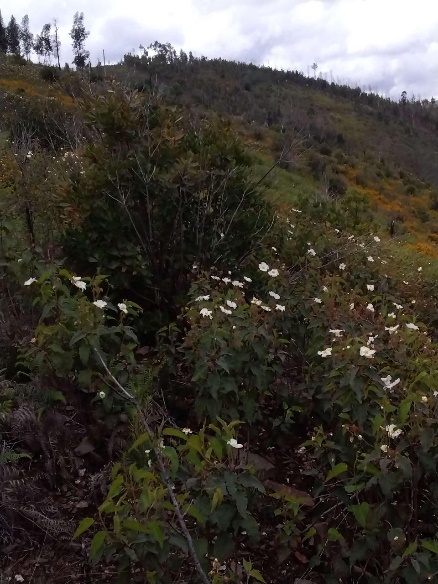  **148** |

| 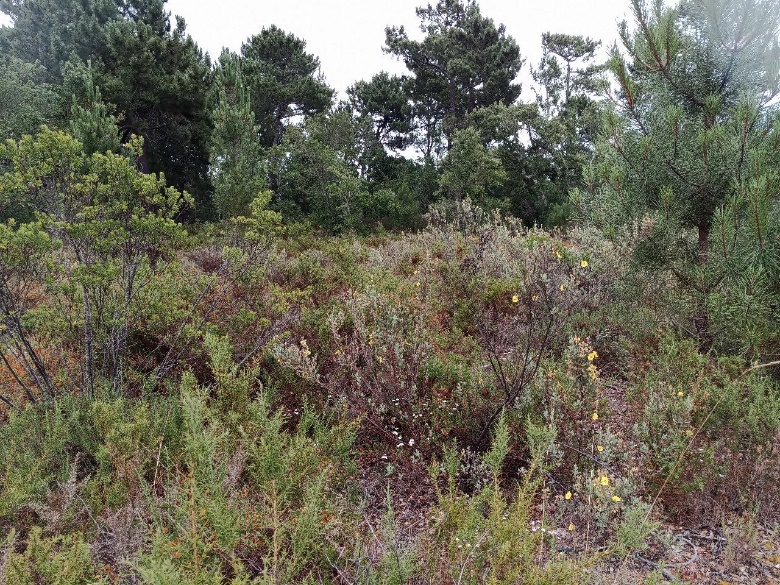  **187** | | 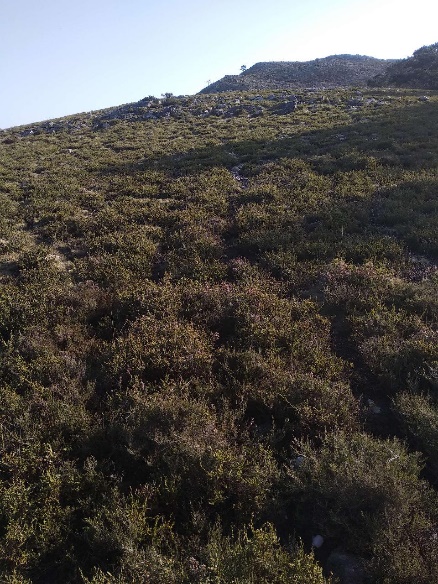  **24** | |
| --- | --- | --- | --- |
|  | |  | |
| 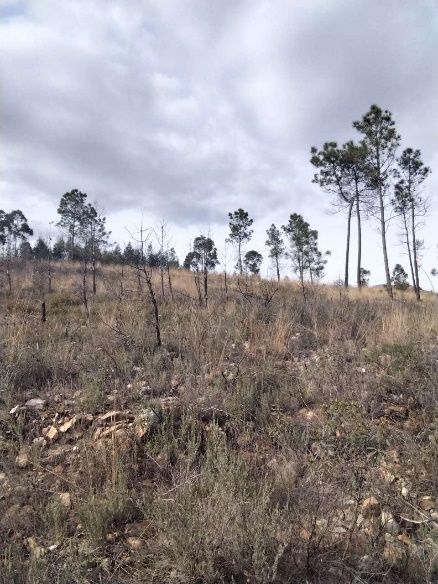  **238** | 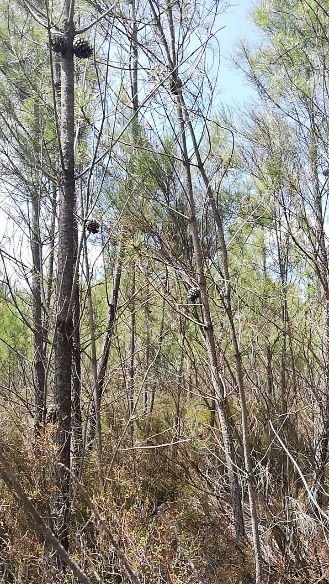  **282** | | 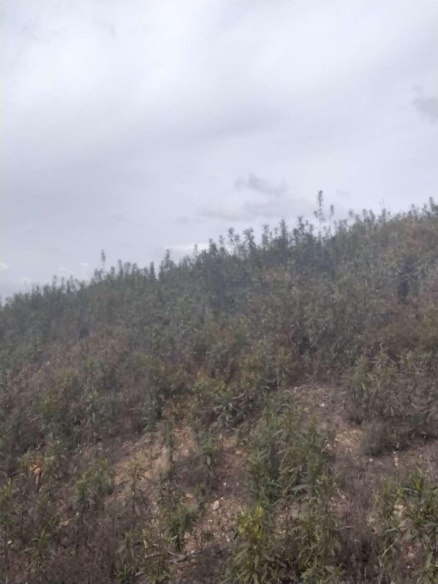  **255** |
|  |  | |  |
| 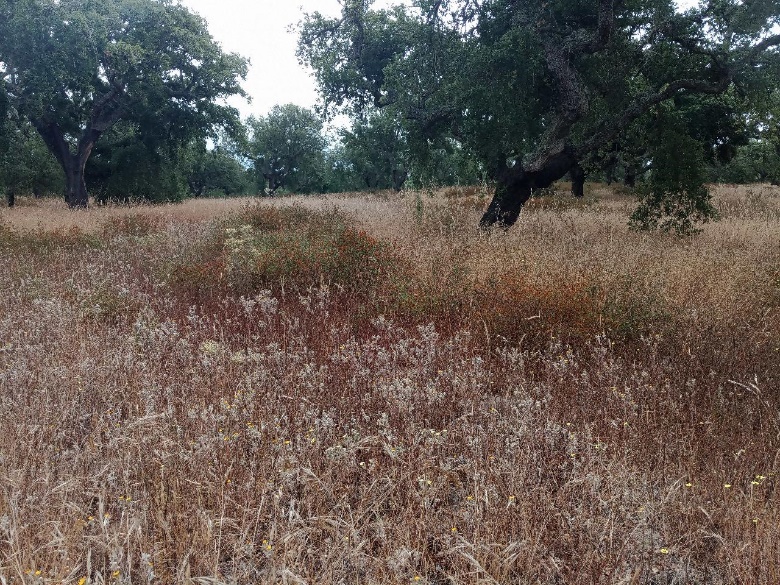  **186** | | 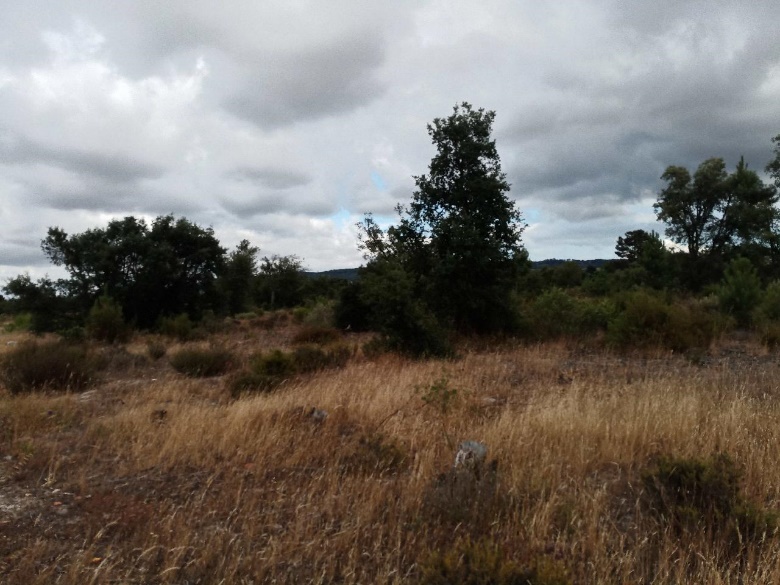  **205** | |
| 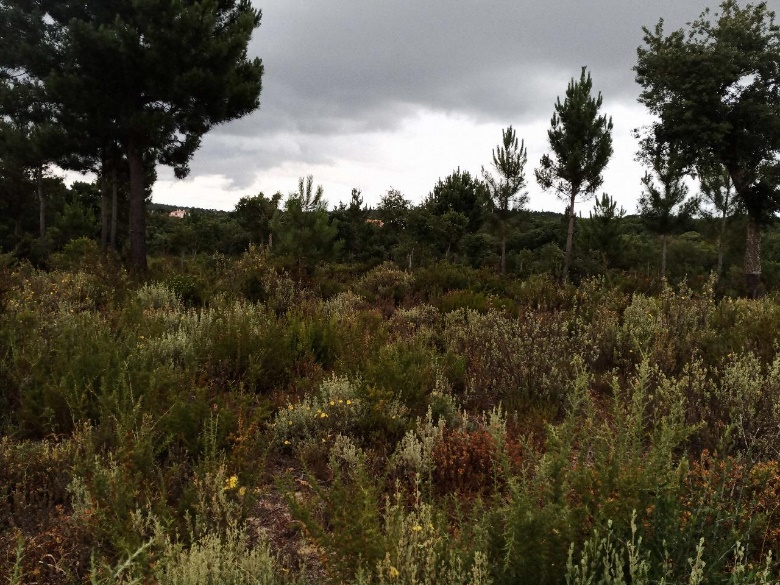  **203** | | 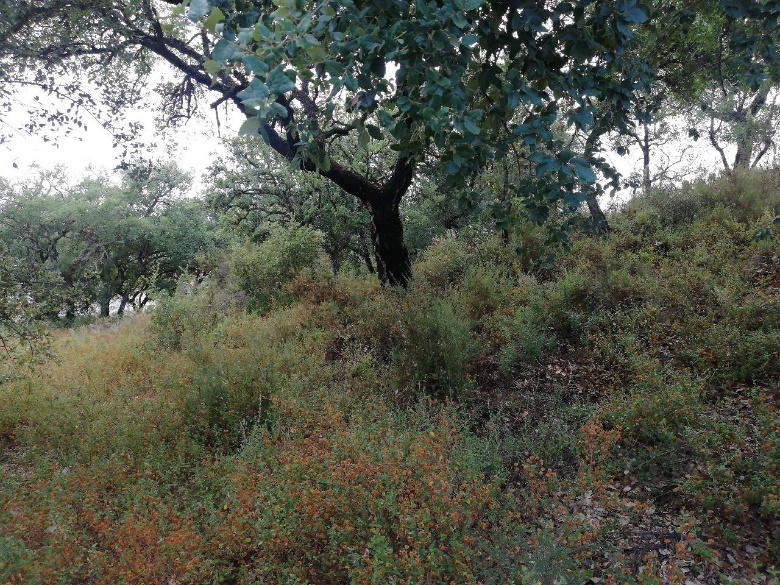  **197** | |
